# New relevant chorological and conservation data on *Carex* (Cyperaceae) and *Hypericum* (Hypericaceae) from Ecuador

**DOI:** 10.3897/BDJ.11.e99603

**Published:** 2023-03-08

**Authors:** Pedro Jiménez-Mejías, Ana Morales-Alonso, Nora H Oleas, Enmily Sánchez, Santiago Martín-Bravo, Irene Masa-Iranzo, Andrea S. Meseguer

**Affiliations:** 1 Department of Biology (Botany), Universidad Autónoma de Madrid, Madrid, Spain Department of Biology (Botany), Universidad Autónoma de Madrid Madrid Spain; 2 Área de Botánica, Department of Molecular Biology and Biochemical Engineering, Universidad Pablo de Olavide, Seville, Spain Área de Botánica, Department of Molecular Biology and Biochemical Engineering, Universidad Pablo de Olavide Seville Spain; 3 Centro de Investigación en Biodiversidad y Cambio Global (CIBC‐UAM), Universidad Autónoma de Madrid, Madrid, Spain Centro de Investigación en Biodiversidad y Cambio Global (CIBC‐UAM), Universidad Autónoma de Madrid Madrid Spain; 4 Centro de Investigación de la Biodiversidad y Cambio Climático (BioCamb) e Ingeniería en Biodiversidad y Recursos Genéticos, Facultad de Ciencias de Medio Ambiente, Universidad Tecnológica Indoamérica, Machala y Sabanilla, Quito, Ecuador Centro de Investigación de la Biodiversidad y Cambio Climático (BioCamb) e Ingeniería en Biodiversidad y Recursos Genéticos, Facultad de Ciencias de Medio Ambiente, Universidad Tecnológica Indoamérica, Machala y Sabanilla Quito Ecuador; 5 Real Jardín Botánico (RJB), CSIC, Madrid, Spain Real Jardín Botánico (RJB), CSIC Madrid Spain

**Keywords:** Andes, chorology, cordillera, endangered species, flora, páramo

## Abstract

**Background:**

Knowledge of *Carex* L. (true sedges) and *Hypericum* L. (St. John's wort) in the Neotropics is fragmentary.

**New information:**

As a result of a fieldwork campaign in Ecuador and revision of herbarium collections (K, QCA and QCNE), we present here relevant records of twelve *Carex* (Cyperaceae) and four *Hypericum* (Hypericaceae) species. Regarding *Carex*, we present the novel report for South America of *C.aztecica*, as well as the first Ecuadorian records for *C.brehmeri*, *C.collumanthus*, *C.fecunda*, *C.melanocystis* and *C.punicola*. The three later records have additional biogeographical significance, as they represent the new northern limit of these species. We also include observations for another five species included in the Ecuadorian Red List of Endemic Plants. As a result, the list of native *Carex* reported for Ecuador would now include 52 taxa. With regard to *Hypericum*, we include the new report of *H.sprucei* for the province of Bolívar, and the confirmation of the presence of three rare species (*H.acostanum*, *H.matangense*, *H.prietoi*) in their type localities, although with extremely low population sizes. We discuss their conservation status and implications.

## Introduction

*Carex* L. (true sedges), is one of the three most diverse plant genera in the World, with more than 2000 species (Roalson et al. 2021). South America harbors about 200 species (Jiménez-Mejías et al. 2018), most of which are endemic to the continent. Some species are dominant in herbaceous habitats, such as bogs or páramos (high altitude Andean grasslands). However and despite its diversity and ecological importance, there is no comprehensive monographic systematic work on South American *Carex*. As a result, as new fieldwork and taxonomic research progresses, new findings, such as new species and chorological records, continue to be published (e.g.Jiménez-Mejías and Escudero (2016), Jiménez-Mejías and Roalson (2016), Poindexter et al. (2017), Jiménez-Mejías and Reznicek (2018), Jiménez-Mejías et al. (2020), Jiménez-Mejías et al. (2021a). Further work is therefore needed to achieve a complete understanding of the taxonomy of these plants in the Neotropics. Ecuador is a diversity hotspot for Neotropical *Carex* (46 spp., of which at least four are endemic to the country: only Argentina and Chile have more *Carex* diversity than Ecuador (Jiménez-Mejías et al. 2018). Ecuadorian *Carex* species are mostly distributed at high altitudes in páramo vegetation, with a few species reaching montane forests at lower altitudes.

With over 600 species, *Hypericum* L. is one of the 100 largest angiosperm genera (Carine and Christenhusz 2013), including economically important species in pharmacology (e.g. *H.perforatum* L., common St. John's wort) and numerous ornamentals. Overall, *Hypericum* has its main centre of species richness in the temperate regions of the Old World (Meseguer et al. 2013), but *Hypericum* species are distributed on almost all continents and radiated in the Neotropics. In South America, *Hypericum* is represented by 102 species (Nürk et al. 2013), most of them endemic and is a prominent component of the páramo shrub flora, but there are also herbaceous species in Brazil and in lowland regions of temperate South America (Robson 1977). Robson published the most complete taxonomic treatment of *Hypericum* to date (Robson 1977, Robson 1981, Robson 1985, Robson 1987, Robson 1990, Robson 1996, Robson 2001, Robson 2002, Robson 2006, Robson 2010a, Robson 2010b, Robson 2012). Since then, new species have being described regularly and chorological information has been updated. However, new fieldwork campaigns are needed to improve our understanding of the diversity and distribution of *Hypericum*. Ecuador is home to 19 species of *Hypericum*, nine of which are endemic to the country (Robson 1987, Robson 1990). The Ecuadorian *Hypericum* species are mainly distributed at high altitudes in páramo vegetation, with few species entering open grassy areas at lower altitudes.

As a result of an international collaboration between the Universidad Autónoma de Madrid (UAM, Madrid, Spain), the Universidad Pablo de Olavide (UPO, Seville, Spain), the Real Jardín Botánico (RJB-CSIC, Madrid, Spain) and the Universidad Tecnológica Indoamérica (UTI, Quito, Ecuador), a fieldwork campaign focusing on *Carex* and *Hypericum* was carried out in the Ecuadorian Andes from Loja to Carchi provinces during the summer of 2022. In addition, the complete *Carex* and *Hypericum* collections of the two main national Ecuadorian herbaria, QCA and QCNE (acronyms according to Thiers (2022)) were studied *in situ*. The present study summarises the main chorological results of this work.

## Materials and methods

The complete *Carex* and *Hypericum* collections of QCA (Herbario de la Pontificia Universidad Católica del Ecuador) and QCNE (Herbario Nacional del Ecuador (QCNE) del Instituto Nacional de Biodiversidad (INABIO), as well as the complete South American *Carex* collection of K (Royal Botanic Gardens, Kew) were studied *in situ*. Herbarium specimens from field collections were deposited at HUTI (Herbario de la Universidad Tecnológica Indoamérica), MA (Herbario del Real Jardín Botánico de Madrid, CSIC) and UPOS (Herbario de la Universidad Pablo de Olavide).

*Carex* specimens were determined using the specialised literature cited for taxon. Species are presented in alphabetical order according to the accepted names provided by Govaerts et al. (2007). The general distribution is also based on Govaerts et al. (2007) and supplemented by the relevant literature cited in each epigraph. Terminology of the inflorescence prophylls (utricles and cladoprophylls) follows suggestions in Jiménez-Mejías et al. (2016). Infrageneric placement follows Roalson et al. (2021) unless otherwise indicated.

*Hypericum* species are listed in alphabetical order according to the accepted names provided by Robson in Studies in the genus *Hypericum* (Robson 1987, Robson 1990). Species distributions follow Robson (Robson 1987, Robson 1990).

Comments for species included in the Red List of the Endemic Plants of Ecuador (León Yánez et al. 2011) are given where relevant. When the Red List includes a name that is no longer in use, it is listed as a synonym under the accepted name.

## Taxon treatments

### Carex acutata

Boott, Proc. Linn. Soc. London 1: 287 (1846)

8163819B-5B69-5882-9A21-19DD7DE9AC7B

=*Carextessellata* Spruce ex C.B.Clarke, Bull. Misc. Inform. Kew, Addit. Ser. 8: 86 (1908).

#### Taxon discussion

*Carexacutata* is a member of the Hirta Clade (subg. Carex), a lineage with its highest diversity in the Holarctic, but with regional diversification in South America (P. Jiménez-Mejías, pers. obs.). It is distributed in montane and high elevation páramo habitats of the Tropical Andes, from Bolivia to Venezuela.

The name *Carextessellata* was included in the Ecuadorian Red List as a “mysterious species known from a single collection of uncertain precedence”. The name was synonymised by Jiménez-Mejías et al. (2020) to *C.acutata* after examining *C.tessellata* type (K-000584703). *Carexacutata* is a species widely distributed in South America and recorded from quite a few locations within Ecuador. Accordingly, the name should be removed from the Red List and the conservation status of *C.acutata* in Ecuador should be re-evaluated.

### Carex aztecica

Mack. in N.L.Britton & al. (eds.), N. Amer. Fl. 18: 229 (1935)

68EF7BFE-923C-50AE-8754-0AE0C069CE1F

#### Materials

- **Type status:** Other material. **Occurrence:** catalogNumber: QCA 36385; recordedBy: S. Lægaard; occurrenceID: QCA 36385; **Taxon:** scientificNameID: *Carexaztecica* Mack; **Location:** country: Ecuador; locality: Cotopaxi. La Maná – Latacunga; verbatimElevation: 1200 m; locationRemarks: Cotopaxi. La Maná – Latacunga km 26, 79°07’W 00°52’S, 1200 m, rocky roadbanks; georeferenceProtocol: label; **Event:** eventDate: 7 Apr 1993
- **Type status:** Other material. **Occurrence:** catalogNumber: QCNE 124910; recordedBy: S. Lægaard; occurrenceID: QCNE 124910; **Taxon:** scientificNameID: *Carexaztecica* Mack; **Location:** country: Ecuador; locality: Cotopaxi. La Maná – Latacunga; verbatimElevation: 1201 m; locationRemarks: Cotopaxi. La Maná – Latacunga km 26, 79°07’W 00°52’S, 1200 m, rocky roadbanks; georeferenceProtocol: label; **Event:** eventDate: 7 Apr 1993

#### Taxon discussion

*Carexaztecica* was hitherto considered a Mesoamerican endemic species, known from southern Mexico and Guatemala. It belongs to the problematic Decora Clade (subg. Carex), where it was included as part of the variation of sect. Indicae Tuckerman *sensu lato*, a set of species with lax paniculate inflorescences and utriculiform cladoprophylls. The Decora clade is one of the two groups of *Carex* that are exclusively distributed in tropical-subtropical areas, along with sect. Fecundae Kük. (see below).

The records that we present here are the first for Ecuador, but also for South America. In addition, they constitute the new known southernmost limit of the species. The taxon can be distinguished from the closely related *C.polystachya* Wahlenb. by its darker female glumes (purple-brown to dark-brown in *C.aztecica* vs. orange, pale reddish-brown, stramineous or hyaline in *C.polystachya*) with blunter apex (at least some glumes obtuse or acute vs. all glumes acuminate to awned) and the utricles with the beak reddish-tinged (vs. not tinged) (Hermann (1974), Chater (1980). These localities in Ecuador become the new known southernmost limit of the species.

### Carex brehmeri

Boeckeler, Allg. Bot. Z. Syst. 2: 190 (1896)

91B060B6-E4AC-5296-A75D-CF749BD7A7B9

#### Materials

- **Type status:** Other material. **Occurrence:** catalogNumber: QCA 238505; recordNumber: 800; recordedBy: S. Salgado; occurrenceID: QCA 238505; **Taxon:** scientificName: *Carexbrehmeri* Boeckeler; **Location:** country: Ecuador; stateProvince: Imbabura; verbatimLocality: Volcán Cotacachi, vía a las lagunas de Piñan; verbatimElevation: 3838 m; locationRemarks: Imbabura. Volcán Cotacachi, vía a las lagunas de Piñán, 3838 m; georeferenceProtocol: label; **Event:** verbatimEventDate: 21 Jan 2009; **Record Level:** basisOfRecord: PreservedSpecimen

#### Taxon discussion

*Carexbrehmeri* is a poorly known Andean taxon, reported from isolated locations in Venezuela, Colombia, Peru and Bolivia (Jiménez-Mejías et al. 2020, Jiménez-Mejías et al., under review). It is closely related to *C.enneastachya* C.B.Clarke (Benítez-Benítez et al. 2021b; see also below). Both species belong to sect. Phacocystis Dumort. (subg. Carex), a subcosmopolitan group with a few South American species.

We provide the first record of this small plant from Ecuador. Despite the fact that it might actually be a mere small form of *C.enneastachya* (see Jiménez-Mejías et al. 2020), we present here *C.brehmeri* separately as a compromise solution to uncover the existence of such taxonomically problematic forms in Ecuador. A comprehensive sampling of the South American species and populations of sect. Phacocystis and a detailed systematic study are needed to unravel the taxonomy of the group in the continent.

### Carex collumanthus

(Steyerm.) L.E.Mora, Acta Biol. Colomb. 1: 40 (1982)

01E35D6C-E498-5942-998B-AFD47D78300E

#### Materials

- **Type status:** Other material. **Occurrence:** catalogNumber: QCA 36562; recordNumber: 705; recordedBy: P. Sklenář & V. Kostečková; occurrenceID: QCA 36562; **Taxon:** scientificName: *Carexcollumanthus* (Steyerm.) L.E.Mora; **Location:** country: Ecuador; stateProvince: Carchi; locality: Chiles volcano; verbatimElevation: 4300-4400 m; locationRemarks: Carchi. Chiles volcano, SW side. 0°48'N 77°57'W, 4300-4400 m, humid superpáramo on shallow sandy soil with rocks; georeferenceProtocol: label; **Event:** eventDate: Jun-21-1995; habitat: humid superpáramo on shallow sandy soil with rocks; **Record Level:** basisOfRecord: PreservedSpecimen
- **Type status:** Other material. **Occurrence:** catalogNumber: QCA 36510; recordNumber: 1045; recordedBy: P. Sklenář & V. Kostečková; occurrenceID: QCA 36510; **Taxon:** scientificName: *Carexcollumanthus* (Steyerm.) L.E.Mora; **Location:** country: Ecuador; stateProvince: Chimborazo; locality: El Altar volcano; verbatimElevation: 4200-4400 m; locationRemarks: Chimborazo. El Altar volcano, N side. 1°41'S 78°24'W, 4200-4400 m, humid cushion superpáramo with scattered bunchgrasses on the ridge below the Canoningo peak; georeferenceProtocol: label; **Event:** eventDate: 19 Aug 1995; habitat: humid cushion superpáramo with scattered bunchgrasses on the ridge below the Canoningo peak; **Record Level:** basisOfRecord: PreservedSpecimen
- **Type status:** Other material. **Occurrence:** catalogNumber: QCA 36525; recordNumber: 2177; recordedBy: P. Sklenář & V. Kostečková; occurrenceID: QCA 36525; **Taxon:** scientificName: *Carexcollumanthus* (Steyerm.) L.E.Mora; **Location:** country: Ecuador; stateProvince: Chimborazo; locality: Chimborazo volcano; verbatimElevation: 4300 m; locationRemarks: Chimborazo. Chimborazo volcano, E side. 01°28'S 78°46'W, 4300 m, superpáramo vegetation along wet gentle slope dominated by cushions of *Werneriahumilis*, *Distichiamuscoides*, *Plantagorigida* and *Azorella* spp.; georeferenceProtocol: label; **Event:** eventDate: Jul-03-1997; **Record Level:** basisOfRecord: PreservedSpecimen
- **Type status:** Other material. **Occurrence:** catalogNumber: QCA 36526; recordNumber: 2177; recordedBy: P. Sklenář & V. Kostečková; occurrenceID: QCA 36526; **Taxon:** scientificName: *Carexcollumanthus* (Steyerm.) L.E.Mora; **Location:** country: Ecuador; stateProvince: Chimborazo; locality: Chimborazo volcano; verbatimElevation: 4300 m; locationRemarks: Chimborazo. Chimborazo volcano, E side. 1°28'S 78°46'W, 4300 m, base of the terminal moraine of the mountain, well developed superpáramo with cushions of *Werneriahumilis*, *Plantagorigida*, *Azorella* spp. and *Azorellaaretioides*; georeferenceProtocol: label; **Event:** eventDate: Jul-03-1997; **Record Level:** basisOfRecord: PreservedSpecimen
- **Type status:** Other material. **Occurrence:** catalogNumber: QCA 36603; recordNumber: 7165; recordedBy: P. Sklenář & V. Kostečková; occurrenceID: QCA 36603; **Taxon:** scientificName: *Carexcollumanthus* (Steyerm.) L.E.Mora; **Location:** country: Ecuador; stateProvince: Chimborazo; locality: Miraloma; locationRemarks: Chimborazo. Miraloma, old road Guamote-Atillo (ca 30 km from Guamote), 02°05'S 78°39'W, transition between grass páramo and superpáramo, 11 Jun 1999, P. Sklenář 7165; georeferenceProtocol: label; **Event:** eventDate: Jun-11-1999; habitat: t; **Record Level:** basisOfRecord: PreservedSpecimen
- **Type status:** Other material. **Occurrence:** catalogNumber: QCA 36602, QCNE 28718; recordNumber: 70550; recordedBy: S. Lægaard; occurrenceID: QCA 36602, QCNE 28718; **Taxon:** scientificName: *Carexcollumanthus* (Steyerm.) L.E.Mora; **Location:** country: Ecuador; stateProvince: Chimborazo; locality: Chimborazo volcano; verbatimElevation: 4800 m; locationRemarks: Chimborazo. Chimborazo volcano, around lower refugio. 01°27'S 78°50'W, 4800 m, dense mats in spring; georeferenceProtocol: label; **Event:** eventDate: Mar-05-1988; habitat: dense mats in spring; **Record Level:** basisOfRecord: PreservedSpecimen
- **Type status:** Other material. **Occurrence:** catalogNumber: QCA 36539; recordNumber: 3038; recordedBy: S. Lægaard; occurrenceID: QCA 36539; **Taxon:** scientificName: *Carexcollumanthus* (Steyerm.) L.E.Mora; **Location:** country: Ecuador; stateProvince: Chimborazo/Morona Santiago; locality: Cerros Yuibug – Pailacajas; verbatimElevation: 4330 m; locationRemarks: Chimborazo/Morona Santiago. Cerros Yuibug – Pailacajas, NE side, 1°45'S 78°27'W, 4300-4330 m, just below vertical rocks, humid upper superpáramo with cushions of Azorella spp., Werneriahumilis, and many mosses; georeferenceProtocol: label; **Event:** eventDate: Jul-31-1997; **Record Level:** basisOfRecord: PreservedSpecimen
- **Type status:** Other material. **Occurrence:** catalogNumber: QCA 36605; recordNumber: 7478; recordedBy: P. Sklenář & V. Kostečková; occurrenceID: QCA 36605; **Taxon:** scientificName: *Carexcollumanthus* (Steyerm.) L.E.Mora; **Location:** country: Ecuador; stateProvince: Cotopaxi; locality: Cotopaxi volcano; verbatimElevation: 4100-4600 m; locationRemarks: Cotopaxi. Cotopaxi volcano, NE side. 00°39'S 78°25'W, 4100-4600 m, superpáramo vegetation; georeferenceProtocol: label; **Event:** eventDate: Jun-28-1999; **Record Level:** basisOfRecord: PreservedSpecimen
- **Type status:** Other material. **Occurrence:** catalogNumber: QCA 36546; recordNumber: 3491; recordedBy: P. Sklenář & V. Kostečková; occurrenceID: QCA 36546; **Taxon:** scientificName: *Carexcollumanthus* (Steyerm.) L.E.Mora; **Location:** country: Ecuador; stateProvince: Napo; locality: Antisana volcano; verbatimElevation: 4200-4300 m; locationRemarks: Napo. Antisana volcano, NE side. 0°27'S 78°08'W, 4200-4300 m, lower superpáramo vegetation along wet rocky escarpments and *Distichiamuscoides* cushion bog; georeferenceProtocol: label; **Event:** eventDate: 19 Aug 1997; **Record Level:** basisOfRecord: PreservedSpecimen
- **Type status:** Other material. **Occurrence:** catalogNumber: QCA 36604; recordNumber: 7286; recordedBy: P. Sklenář & V. Kostečková; occurrenceID: QCA 36604; **Taxon:** scientificName: *Carexcollumanthus* (Steyerm.) L.E.Mora; **Location:** country: Ecuador; stateProvince: Napo; locality: Páramo de Guamaní; verbatimElevation: 4300-4370 m; locationRemarks: Napo. Páramo de Guamaní. 00°19'S 78°11'W, 4300-4370 m, humid superpáramo; georeferenceProtocol: label; **Event:** eventDate: Jun-19-1999; habitat: humid superpáramo; **Record Level:** basisOfRecord: PreservedSpecimen
- **Type status:** Other material. **Occurrence:** catalogNumber: QCA 36601; recordNumber: 70500; recordedBy: S. Lægaard; occurrenceID: QCA 36601; **Taxon:** scientificName: *Carexcollumanthus* (Steyerm.) L.E.Mora; **Location:** country: Ecuador; stateProvince: Pichincha; locality: Cayambe volcano; verbatimElevation: 4400-4500 m; locationRemarks: Pichincha. Cayambe volcano, along road to Refugio. 00°04'S 77°57'W, 4400-4500 m, in dense mats of *Distichiamuscoides*; georeferenceProtocol: label; **Event:** eventDate: Mar-01-1988; **Record Level:** basisOfRecord: PreservedSpecimen
- **Type status:** Other material. **Occurrence:** catalogNumber: QCA 36542; recordNumber: 344; recordedBy: P. Sklenář & V. Kostečková; occurrenceID: QCA 36542; **Taxon:** scientificName: *Carexcollumanthus* (Steyerm.) L.E.Mora; **Location:** country: Ecuador; stateProvince: Pichincha; locality: Rucu Pichincha; verbatimElevation: 4500 m; locationRemarks: Pichincha. Rucu Pichincha volcano. 0°10'S 78°34'W, 4500 m, NE slope of the volcano, superpáramo vegetation on steep sandy slopes and rocks; georeferenceProtocol: label; **Event:** eventDate: May-18-1995; **Record Level:** basisOfRecord: PreservedSpecimen
- **Type status:** Other material. **Occurrence:** catalogNumber: QCA 36600; recordNumber: 51313; recordedBy: S. Lægaard; occurrenceID: QCA 36600; **Taxon:** scientificName: *Carexcollumanthus* (Steyerm.) L.E.Mora; **Location:** country: Ecuador; stateProvince: Pichincha; locality: Páramo Guamaní; verbatimElevation: 4050 m; locationRemarks: Pinchincha. Páramo Guamaní, close to Páramo de la Virgen. 0°20'S 78°13'W, 4050 m, moist páramo and springbogs; georeferenceProtocol: label; **Event:** eventDate: Feb-08-1984; habitat: moist páramo and springbogs; **Record Level:** basisOfRecord: PreservedSpecimen

#### Taxon discussion

*Carexcollumanthus* is endemic from the northern half of the cordillera, where it has been reported from Venezuela, Colombia, Peru and Bolivia (Wheeler 2002). It belongs to sect. Abditispicae G. A. Wheeler, a group of species with Andean-Patagonian distribution that is nested within the Flacca Clade (subg. Carex).

Here, we report the species for the first time in Ecuador, which was somehow expected since the plant is known from Colombia and Peru.

### Carex ecuadorensis

(G.A.Wheeler & Goetgh.) J.R.Starr, Bot. J. Linn. Soc. 179: 31 (2015)

455DACA7-8D99-5206-89A7-322C72AA9121

=*Unciniaecuadorensis* G.A.Wheeler & Goetgh., Aliso 15: 10 (1997).

#### Materials

- **Type status:** Other material. **Occurrence:** catalogNumber: 117ECU-AMA22 (HUTI, UPOS); recordNumber: 117; recordedBy: A. Morales Alonso, P. Jiménez Mejías, I. Masa Iranzo & E. Sánchez; occurrenceID: 117ECU-AMA22 (HUTI, UPOS); **Taxon:** scientificName: *Carexecuadorensis* (G.A.Wheeler & Goetgh.) J.R.Starr; **Location:** country: Ecuador; stateProvince: Imbabura; locality: National Park Cotacachi; verbatimElevation: 3918 m; locationRemarks: Imbabura. Parque Nacional de Cotacachi, camino de las antenas del Cotacachi, 00°19.7943'N 078°20.5391'W, 3918 m, acequia en pajonal, cerca del desagüe al camino; georeferenceProtocol: GPS; **Identification:** identifiedBy: P. Jiménez-Mejías; dateIdentified: 2022; **Record Level:** basisOfRecord: PreservedSpecimen

#### Taxon discussion

**Figure 1.**
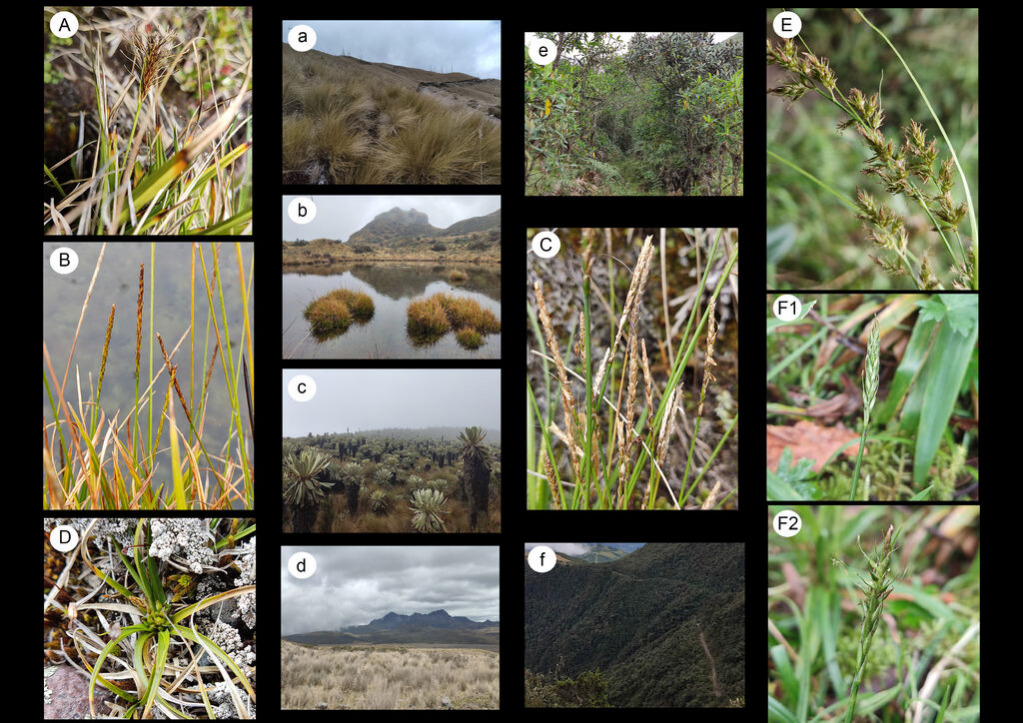
Representative photos of *Carex* species (capital letters) mentioned in this work and their habitats (small letters). *Carexecuadorensis*: (**A,a**), Imbabura, Parque Nacional de Cotacachi, type locality. *Carexmadida*: (**B,b**), Pichincha, Parque Nacional Cayambe-Coca, type locality; (**C,c**), Carchi, Reserva Ecológica El Ángel. *Carexpunicola*: (**D,d**), Pichincha, Parque Nacional Cotopaxi. *Carexsodiroi*: (**E,e**), Pichincha, Reserva Geobotánica Pululahua. *Carexsubsacculata*: (**F1,F2,f**), Pichincha, Reserva Biológica Yanacocha; the specimen in picture F1 is still inmature, while the one in picture F2 is fully ripe.

An Ecuadorian endemic that belongs to sect. Uncinia (Pers.) Baill. (subg. Uncinia (Pers.) Peterm.), a mainly Southern Hemisphere group disjunctly distributed between the Neotropic and the SW Pacific. Formerly recognized as its own genus (*Uncinia* Pers.; Global Carex Group (2015)), it is one of the few *Carex* groups with an unequivocal epizoochorus adaptation: the utricles bear a hooked appendix that allows them to attach to fur or feathers (Fig. 1 A,a).

*Carexecuadorensis* was reported as threatened in the Ecuadorian Red List (as *Unciniaecuadorensis*) under the category vulnerable (VU). Known from only two localities collected in the 80s, we confirm its persistence in at least one of these (the type location) and provide an exact location with coordinates. The reported population was found in a natural drainage channel of the pajonal. It was healthy, with about twenty individuals in good conditions. The only direct threat we noticed is the erosion of the slopes next to the road, which was more developed in other sectors. Probably, *Carexecuadorensis* is growing in similar habitats in additional places of more difficult access within the same area. In any case, the location of this population in a National Park is an important safeguard for the future persistence of this species.

### Carex enneastachya

C.B.Clarke, Bull. Misc. Inform. Kew, Addit. Ser. 8: 70 (1908)

C4A57B85-01B0-50AF-84CE-3EA9CFA1A407

= *Carexazuayae* Steyerm., Phytologia 9: 337 (1963).

#### Materials

- **Type status:** Other material. **Occurrence:** catalogNumber: QCA 36315, QCNE 3907; recordNumber: 2279; recordedBy: H. Balslev; occurrenceID: QCA 36315, QCNE 3907; **Taxon:** scientificName: *Carexenneastachya* C.B.Clarke; **Location:** country: Ecuador; stateProvince: Cotopaxi; locality: National Park Cotopaxi; verbatimLocality: Limpiopungo; verbatimElevation: 3800 m; locationRemarks: Cotopaxi. Parque Nacional Cotopaxi, Limpiopungo, 0°40’S 78°0'W, 3800 m, laguna y alrededores; georeferenceProtocol: label; **Record Level:** basisOfRecord: PreservedSpecimen
- **Type status:** Other material. **Occurrence:** catalogNumber: 88ECU-AMA22 (HUTI, UPOS); recordNumber: 88; recordedBy: A. Morales Alonso, P. Jiménez Mejías & I. Masa Iranzo; occurrenceID: 88ECU-AMA22 (HUTI, UPOS); **Taxon:** scientificName: *Carexenneastachya* C.B.Clarke; **Location:** country: Ecuador; stateProvince: Pichincha; locality: National Park Cotopaxi; verbatimLocality: Laguna Limpiopungo; verbatimElevation: 3864 m; locationRemarks: Pichincha. Laguna Limpiopungo, Parque Nacional Cotopaxi, 00°36.8748'S 078°25.4262'W, 3864 m, bordes de la laguna y arroyos; georeferenceProtocol: GPS; **Identification:** identifiedBy: P. Jiménez-Mejías; dateIdentified: 2022; **Record Level:** basisOfRecord: PreservedSpecimen
- **Type status:** Other material. **Occurrence:** catalogNumber: 98ECU-AMA22 (UPOS); recordNumber: 98; recordedBy: A. Morales Alonso, P. Jiménez Mejías & I. Masa Iranzo; occurrenceID: 98ECU-AMA22 (UPOS); **Taxon:** scientificName: *Carexenneastachya* C.B.Clarke; **Location:** country: Ecuador; stateProvince: Pichincha; locality: National Park Cayambe-Coca; verbatimLocality: Laguna inferior; verbatimElevation: 4117 m; locationRemarks: Pinchincha. Laguna inferior. Parque Nacional Cayambe-Coca, 00°19.6955'S 078°12.0338'W, 4117 m, turbera; georeferenceProtocol: GPS; **Identification:** identifiedBy: P. Jiménez-Mejías; dateIdentified: 2022; **Record Level:** basisOfRecord: PreservedSpecimen
- **Type status:** Other material. **Occurrence:** catalogNumber: QCA 36663; recordNumber: 315; recordedBy: E. Terneus & P. Ramsay; occurrenceID: QCA 36663; **Taxon:** scientificName: *Carexenneastachya* C.B.Clarke; **Location:** country: Ecuador; stateProvince: Tungurahua; locality: National Park Llanganates; verbatimLocality: laguna Aucacocha; verbatimElevation: 3750 m; locationRemarks: Tungurahua. Parque Nacional Llanganates, laguna Aucacocha, 01°09’36’’S 78°19’37’’W, 3750 m, orillas de la laguna; georeferenceProtocol: label; **Event:** eventDate: 13 Jan 1999; **Record Level:** basisOfRecord: PreservedSpecimen

#### Taxon discussion

*Carexenneastachya* is endemic from the Tropical Andes from Colombia to Bolivia. As *C.brehmeri*, it belongs to sect. Phacocystis Dumort. (see above).

In Ecuador, *Carexenneastachya* was reported only from a handful locations in Cajas National Park at Azuay Province. Here we present additional records for another three provinces. Originally conceived as a narrowly distributed taxon, this species was listed as EN in the Ecuadorian Red List of Endemic plants under the name *C.azuayae*. However, *C.azuayae* was already synonymized by Wheeler (1998) to *C.enneastachya*, which effectively expands its range to other South American countries. As a consequence, this plant should not be listed as an Ecuadorian endemic and its threatened status needs revision.

### Carex fecunda

Steud., Syn. Pl. Glumac. 2: 194 (1855)

0C1168EF-E48C-5B3E-8384-F6CC07EA242D

#### Materials

- **Type status:** Other material. **Occurrence:** catalogNumber: 57ECU-AMA22 (HUTI, UPOS); recordNumber: 57; recordedBy: A. Morales Alonso, P. Jiménez Mejías & I. Masa Iranzo; occurrenceID: 57ECU-AMA22 (HUTI, UPOS); **Taxon:** scientificName: *Carexfecunda* Steud.; **Location:** country: Ecuador; stateProvince: Azuay; locality: National Park Cajas; verbatimLocality: Laguna de Llaviucu; verbatimElevation: 3156 m; locationRemarks: Azuay. Laguna de Llaviucu, Parque Nacional Cajas, 02°05.6385'S 079°08.9762'W, 3156 m, pastizales húmedos en el borde de laguna; georeferenceProtocol: GPS; **Identification:** identifiedBy: P. Jiménez-Mejías; dateIdentified: 2022; **Event:** eventDate: Jul-31-2022; **Record Level:** basisOfRecord: PreservedSpecimen

#### Taxon discussion

A South American endemic previously reported from the Central Andes, from Peru to northern Argentina. It belongs to the problematic sect. Fecundae (subg. Carex), a group of sedges entirely diversified within the Neotropic and one of the only two groups of *Carex* exclusively distributed in tropical-subtropical areas (the other sect. Indicae, see above).

This is the first record for Ecuador and the new northernmost limit for the species. The Ecuadorian specimen had the unusual feature of emitting a profound citric scent from the leaves and stems when crushed that persisted after drying. This aromatic property is known from other sedge groups (e.g. *Cyperusodoratus* L., *C.sesquiflorus* (Torr.) Mattf. & Kük.), but it has not been previously reported in *Carex*, including other fresh or dry *C.fecunda* specimens (P. Jiménez-Mejías, pers. obs.). This may indicate some differences with respect to other more southern *C.fecunda* populations and deserves further study.

### Carex goetghebeurii

J.R.Starr, Bot. J. Linn. Soc. 179: 32 (2015)

7EF0EDBB-53BF-5E72-8C08-32307FA090DD

=*Unciniatenuifolia* G.A.Wheeler & Goetgh., Aliso 14: 144 (1995).

#### Taxon discussion

Another Ecuadorian endemic that belongs to sect. Uncinia (see *C.ecuadorensis*).

*Carexgoetghebeurii* was reported as threatened (VU category) in the Ecuadorian Red List (as *Unciniatenuifolia*). This was based on several collections of the same single population extending along a slope next to a road in Zamora-Chinchipe. We visited one of them (“13 km E of the pass, just before junction with old road”, -3.9764109958906992 S, -79.10201083174987 W) and failed to find it. The location has been transformed into a quarry. This unfortunate finding depicts that this plant is already negatively affected by human activities and reveals the need to confirm the persistence of this population and, if positive, to apply urgent conservation measures.

### Carex lepida

Boott, Ill. Gen. Carex 4: 211 (1867)

9397CEA7-763F-517C-BBED-6FCA43995D58

#### Materials

- **Type status:** Other material. **Occurrence:** catalogNumber: 54ECU-AMA22 (HUTI, UPOS); recordNumber: 54; recordedBy: A. Morales Alonso, P. Jiménez Mejías, I. Masa Iranzo & E. Sánchez; occurrenceID: 54ECU-AMA22 (HUTI, UPOS); **Taxon:** scientificName: *Carexlepida* Boott; **Location:** country: Ecuador; stateProvince: Azuay; locality: National Park Cajas; verbatimLocality: Laguna de Llaviucu; verbatimElevation: 3156 m; locationRemarks: Azuay. Laguna de Llaviucu, Parque Nacional Cajas, 02°05.6385'S 079°08.9762'W, 3156 m, borde de camino en bosque montano; georeferenceProtocol: GPS; **Identification:** identifiedBy: P. Jiménez-Mejías; dateIdentified: 2022; **Event:** eventDate: Jul-31-2022; **Record Level:** basisOfRecord: PreservedSpecimen
- **Type status:** Other material. **Occurrence:** catalogNumber: QCA 36473; recordNumber: 45617; recordedBy: B. Boysen, B. Eriksen, L.P. Kvist, D. Nissen & J. Korning; occurrenceID: QCA 36473; **Taxon:** scientificName: *Carexlepida* Boott; **Location:** country: Ecuador; stateProvince: Imbabura; verbatimLocality: Southwestern slopes of the volcano Cotacachi, near the road from Cotacachi to Selva Alegre; verbatimElevation: 3300-3350 m; locationRemarks: Imbabura. Southwestern slopes of the volcano Cotacachi, near the road from Cotacachi to Selva Alegre, 0°21'N 78°26'W, 3300-3350 m, secondary páramo with patches of mountain forest; georeferenceProtocol: label; **Event:** eventDate: Nov-07-1983; habitat: secondary páramo with patches of mountain forest; **Record Level:** basisOfRecord: PreservedSpecimen
- **Type status:** Other material. **Occurrence:** catalogNumber: QCA 36498, QCNE 173344; recordNumber: 1197; recordedBy: B. Øllgaard; occurrenceID: QCA 36498, QCNE 173344; **Taxon:** scientificName: *Carexlepida* Boott; **Location:** country: Ecuador; stateProvince: Imbabura; verbatimLocality: Road Otavalo-Lagunas de Mojanda; verbatimElevation: 3500 m; locationRemarks: Imbabura. Road Otavalo-Lagunas de Mojanda, 0°14’N 78°20’W, 3500 m, disturbed mossy forest; georeferenceProtocol: label; **Event:** eventDate: Oct-21-1995; habitat: disturbed mossy forest; **Record Level:** basisOfRecord: PreservedSpecimen
- **Type status:** Other material. **Occurrence:** catalogNumber: QCA 36435; recordNumber: 54171; recordedBy: S. Lægaard; occurrenceID: QCA 36435; **Taxon:** scientificName: *Carexlepida* Boott; **Location:** country: Ecuador; stateProvince: Pichincha; locality: Volcán Pasochoa; verbatimLocality: above house of Fundación Natura; verbatimElevation: 2800-3300 m; locationRemarks: Pichincha. Volcán Pasochoa, above house of Fundación Natura, 0°27’S 78°31’W, 2800-3300 m, grass-field, mountain forest and páramo; georeferenceProtocol: label; **Event:** eventDate: 27 Apr 1985; habitat: grass-field, mountain forest and páramo; **Record Level:** basisOfRecord: PreservedSpecimen
- **Type status:** Other material. **Occurrence:** catalogNumber: QCA 36379, QCNE 172674; recordNumber: 101411; recordedBy: S. Lægaard; occurrenceID: QCA 36379, QCNE 172674; **Taxon:** scientificName: *Carexlepida* Boott; **Location:** country: Ecuador; stateProvince: Pichincha; verbatimLocality: Refugio de Vida Silvestre Pasochoa; verbatimElevation: 3100-3400 m; locationRemarks: Pichincha. Refugio de Vida Silvestre Pasochoa, 0°21’S 78°29’W, 3100-3400 m; georeferenceProtocol: label; **Event:** eventDate: Feb-23-1992; habitat: bosque nublado con un estrato bajo muy denso; **Record Level:** basisOfRecord: PreservedSpecimen
- **Type status:** Other material. **Occurrence:** catalogNumber: QCA 36680, QCNE 154445; recordNumber: s.n; recordedBy: R.Bu & SL; occurrenceID: QCA 36680, QCNE 154445; **Taxon:** scientificName: *Carexlepida* Boott; **Location:** country: Ecuador; stateProvince: Zamora-Chinchipe; locality: Cordillera de Sabanilla; verbatimLocality: cerca de la carretera Jimbura-Zumba; verbatimElevation: 3100 m; locationRemarks: Zamora-Chinchipe. Cordillera de Sabanilla, cerca de la carretera Jimbura-Zumba, 04°42’41''S 79°26’25''W, 3100 m, bosque nublado con un estrato bajo muy denso; georeferenceProtocol: label; **Event:** eventDate: Oct-23-1996; **Record Level:** basisOfRecord: PreservedSpecimen

#### Taxon discussion

*Carexlepida* was described in the 19^th^ Century from Pichincha Province and remained known only from the type location until additional records were recently reported from Ecuador, Bolivia and Peru (Jiménez-Mejías and Escudero (2016), Jiménez-Mejías and Reznicek (2018), Jiménez-Mejías et al., under review). Its systematic affinities have long remained enigmatic until it was recently placed in sect. Wheelerianae Jim.-Mejías, Martín-Bravo and Reznicek (subg. Uncinia), based on molecular phylogenetics. It is a South American endemic lineage from Andean montane forests (García-Moro et al. 2022).

Remarkably, the species was listed in the Ecuadorian Red List of Endemic plants as critically endangered (CR), as it was known only from the type location from Pichincha volcano slopes, making its persistence there improbable due to the urban growth of Quito. A previous report of a recent collection also from Pichincha already existed (Pasochoa volcano; Jiménez-Mejías and Reznicek (2018)). Our records constitute a considerable extension of the known range of the species within Ecuador. According to all the new available data, the conservation status of the species should be revised. Additionally, it should no longer be considered endemic to Ecuador.

### Carex lepida × roalsoniana

28E40763-5517-5DB4-8039-8CC20446A2C0

#### Materials

- **Type status:** Other material. **Occurrence:** catalogNumber: 55bECU-AMA22 (HUTI, UPOS); recordNumber: 55b; recordedBy: A. Morales Alonso, P. Jiménez Mejías, I. Masa Iranzo & E. Sánchez; occurrenceID: 55bECU-AMA22 (HUTI, UPOS); **Taxon:** scientificName: *Carexlepida* × *Carexroalsoniana*; **Location:** country: Ecuador; stateProvince: Azuay; locality: National Park Cajas; verbatimLocality: Laguna de Llaviucu; verbatimElevation: 3156 m; locationRemarks: Azuay. Laguna de Llaviucu, Parque Nacional Cajas, 02°05.6385'S 079°08.9762'W, 3156 m, borde de camino en bosque montano; georeferenceProtocol: GPS; **Identification:** identifiedBy: P. Jiménez-Mejías; dateIdentified: 2022; **Event:** eventDate: Jul-31-2022; **Record Level:** basisOfRecord: PreservedSpecimen

#### Taxon discussion

First insights of hybridization amongst species of sect. Wheelerianae. The sampled specimens were co-occurring with the two putative parental species in the area (see comments under *C.lepida* and *C.roalsoniana*) and showed intermediate morphological characteristics between them (see Jiménez-Mejías and Reznicek (2018)).

### Carex madida

J.R.Starr, Bot. J. Linn. Soc. 179: 34 (2015)

7E6166D7-67AF-5A69-99F8-36A7D5216DC3

=*Uncinialacustris* G.A.Wheeler, Aliso 14: 141 (1995).

#### Materials

- **Type status:** Other material. **Occurrence:** catalogNumber: 110 ECU-AMA22 (HUTI, UPOS); recordNumber: 110; recordedBy: Morales Alonso, A., Jiménez Mejías, P., Masa Iranzo, I & Sánchez, E.; occurrenceID: 110 ECU-AMA22 (HUTI, UPOS); **Taxon:** scientificName: *Carexmadida* J.R.Starr; **Location:** country: Ecuador; stateProvince: Carchi; locality: Reserva Ecológica El Ángel; verbatimLocality: carretera a las Lagunas Verdes del Volcán Chiles; locationRemarks: Carchi. Reserva Ecológica El Ángel, carretera a las Lagunas Verdes del Volcán Chiles. 00°47.3118'N 077°53.5117W, 3682 m, borde de camino en pajonal con formaciones de *Espeletia* spp.; georeferenceProtocol: GPS; **Identification:** identifiedBy: P. Jiménez-Mejías; dateIdentified: 2022; **Event:** eventDate: 6 Aug 2022; **Record Level:** basisOfRecord: PreservedSpecimen
- **Type status:** Other material. **Occurrence:** catalogNumber: QCA221830; recordNumber: 166; recordedBy: V. Yunapanta & S. Chimbolema; occurrenceID: QCA221830; **Taxon:** scientificName: *Carexmadida* J.R.Starr; **Location:** country: Ecuador; stateProvince: Carchi; locality: Reserva Ecológica El Ángel; verbatimLocality: Cantones de Tulcán, Espejo y Mira, en la zona de amortiguamiento de la Reserva Ecológica El Ángel; locationRemarks: Carchi. Cantones de Tulcán, Espejo y Mira, en la zona de amortiguamiento de la Reserva Ecológica El Ángel. 00°40'N 77°51W, 3635 m, bosque siempreverde montano alto y páramo de frailejones; georeferenceProtocol: label; **Event:** eventDate: Oct-08-2011; **Record Level:** basisOfRecord: PreservedSpecimen
- **Type status:** Other material. **Occurrence:** catalogNumber: QCA 221804; recordNumber: 397; recordedBy: H. Valles & S. Chimbolema; occurrenceID: QCA 221804; **Taxon:** scientificName: *Carexmadida* J.R.Starr; **Location:** country: Ecuador; stateProvince: Carchi; locality: Reserva Ecológica El Ángel; verbatimLocality: Cantón Espejo, parroquia de la Libertad; verbatimElevation: 3781 m; locationRemarks: Carchi. Cantón Espejo, parroquia de la Libertad, Reserva Ecológica El Ángel. 00°42'N, 77°52'W, 3781 m,; georeferenceProtocol: label; **Event:** eventDate: Jun-20-2011; **Record Level:** basisOfRecord: PreservedSpecimen
- **Type status:** Other material. **Occurrence:** catalogNumber: QCA 38381; recordNumber: 386; recordedBy: P. Ramsay & P.J. Merrow-Smith; occurrenceID: QCA 38381; **Taxon:** scientificName: *Carexmadida* J.R.Starr; **Location:** country: Ecuador; stateProvince: Chimborazo; locality: Collanes Valley, Páramo de los Altares; locationRemarks: Chimborazo. Collanes Valley, Páramo de los Altares, 01°40'S 78°24'W, 3900 m, páramo grassland; georeferenceProtocol: label; **Event:** eventDate: Sep-03-1987; **Record Level:** basisOfRecord: PreservedSpecimen
- **Type status:** Other material. **Occurrence:** catalogNumber: 118 ECU-AMA22 (HUTI, UPOS); recordNumber: 118; recordedBy: Morales Alonso, A., Jiménez Mejías, P., Masa Iranzo, I & Sánchez, E.; occurrenceID: 118 ECU-AMA22 (HUTI, UPOS); **Taxon:** scientificName: *Carexmadida* J.R.Starr; **Location:** country: Ecuador; stateProvince: Imbabura; locality: National Park Cotacachi-Cayapas; locationRemarks: Imbabura. Parque Nacional Cotacachi-Cayapas, 00°19.79'N 078°20.53'W, 3918 m, acequia en pajonal, cerca del camino; georeferenceProtocol: GPS; **Identification:** identifiedBy: P. Jiménez-Mejías; dateIdentified: 2022; **Event:** eventDate: 7 Aug 2022; **Record Level:** basisOfRecord: PreservedSpecimen
- **Type status:** Other material. **Occurrence:** catalogNumber: 96ECU-AMA22 (HUTI, UPOS); recordNumber: 96; recordedBy: Morales Alonso, A., Jiménez Mejías, P., Masa Iranzo, I & Sánchez, E.; occurrenceID: 96ECU-AMA22 (HUTI, UPOS); **Taxon:** scientificName: *Carexmadida* J.R.Starr; **Location:** country: Ecuador; stateProvince: Pichincha; locality: National Park Cayambe-Coca; verbatimLocality: orillas de una pequeña laguna; locationRemarks: Pichincha. Parque Nacional Cayambe-Coca, 00°19.4672'S 078°12.0532'W, 4167 m; georeferenceProtocol: GPS; **Identification:** identifiedBy: P. Jiménez-Mejías; dateIdentified: 2022; **Event:** eventDate: 4 Aug 2022; **Record Level:** basisOfRecord: PreservedSpecimen
- **Type status:** Other material. **Occurrence:** catalogNumber: 99ECU-AMA22 (UPOS); recordNumber: 99; recordedBy: Morales Alonso, A., Jiménez Mejías, P., Masa Iranzo, I & Sánchez, E.; occurrenceID: 99ECU-AMA22 (UPOS); **Taxon:** scientificName: *Carexmadida* J.R.Starr; **Location:** country: Ecuador; stateProvince: Pichincha; locality: National Park Cayambe-Coca; verbatimLocality: en turbera; locationRemarks: Pichincha. Parque Nacional Cayambe-Coca, 00°19.6955'S 078°12.0338'W, 4117 m, en turbera; georeferenceProtocol: GPS; **Identification:** identifiedBy: P. Jiménez-Mejías; dateIdentified: 2022; **Event:** eventDate: 4 Aug 2022; **Record Level:** basisOfRecord: PreservedSpecimen
- **Type status:** Other material. **Occurrence:** catalogNumber: QCA 225822; recordNumber: 5799; recordedBy: K. Romoleroux & G. Peyre; occurrenceID: QCA 225822; **Taxon:** scientificNameID: *Carexmadida* J.R.Starr; **Location:** country: Ecuador; stateProvince: Carchi; locality: Tulcán, Páramo de El Ángel; verbatimElevation: 3778 m; locationRemarks: Tulcán, Páramo de El Ángel, 3778 m; georeferenceProtocol: label; **Record Level:** collectionID: QCA 225822

#### Taxon discussion

This species belongs to sect. Uncinia (see *C.ecuadorensis*), which remarkably includes three of the four Ecuadorian endemic *Carex* species (Fig. 1B,b,C,c).

*Carexmadida* was reported as vulnerable (VU) in the Ecuadorian Red List (as *Uncinialacustris*), based on the known existence of only three localities, all in protected areas (Cayambe-Coca, Cotacachi-Cayapas and Cotopaxi National Parks) in the provinces of Pichincha, Napo and Imbabura, respectively. Here, we report its presence in two additional provinces (Carchi and Chimborazo) and one addition to a protected area (Reserva Ecológica El Ángel). We confirm its persistence in two of the formerly known localities, where it seems abundant. Since the plants occur in *Espeletia* formations very close to the Colombian border, it is very likely that this species also occurs in the neighbouring country, from which it has not been reported yet.

### Carex melanocystis

É.Desv. in C.Gay, Fl. Chil. 6: 203 (1854)

FCB308FB-AC29-5774-A6FD-08DCA35B7E3A

#### Materials

- **Type status:** Other material. **Occurrence:** catalogNumber: QCA 36444, QCNE 141330; recordNumber: 55347; recordedBy: S. Lægaard; occurrenceID: QCA 36444, QCNE 141330; **Taxon:** scientificName: *Carexmelanocystis* É.Desv; **Location:** country: Ecuador; stateProvince: Bolívar; verbatimLocality: Km 4 on road Los Arenales-Salinas; verbatimElevation: 4300 m; locationRemarks: Bolívar. Km 4 on road Los Arenales-Salinas, 01°24’S 78°55’W, 4300 m, grass-sward; georeferenceProtocol: label; **Event:** eventDate: Oct-02-1985; habitat: grass-sward; **Record Level:** basisOfRecord: PreservedSpecimen

#### Taxon discussion

*Carexmelanocystis* is widely distributed across the Andes, from Tierra del Fuego to the Central Andes of Peru (Jiménez-Mejías et al. 2020, Jiménez-Mejías et al. 2021a). It has been recently segregated from the closely-related Holarctic *C.maritima* Gunnerus (Jiménez-Mejías et al. 2021b). *Carexmelanocystis* is the only Southern Hemisphere representative of the Disticha clade (subg. Vignea (T.Lestib) Peterm.), where both species are nested.

These are the first confirmed records of *C.melanocystis* for Ecuador, which become the northernmost limit of the species.

### Carex phalaroides

Kunth, Enum. Pl. 2: 482 (1837)

FA19551E-E455-5C30-A981-7CA28DE8B377

#### Materials

- **Type status:** Other material. **Occurrence:** catalogNumber: QCA 36470; recordNumber: 71694; recordedBy: S. Lægaard; occurrenceID: QCA 36470; **Taxon:** scientificName: *Carexphalaroides* Kunth; **Location:** country: Ecuador; stateProvince: Chimborazo; locality: Arenales de Palmira; verbatimElevation: 3300 m; locationRemarks: Chimborazo. Arenales de Palmira, 02°02’S 78°45’W, 3300 m; georeferenceProtocol: label; **Event:** eventDate: Jun-26-1988; **Record Level:** basisOfRecord: PreservedSpecimen
- **Type status:** Other material. **Occurrence:** catalogNumber: QCNE82136; recordNumber: 106; recordedBy: M. Garrison; occurrenceID: QCNE82136; **Taxon:** scientificName: *Carexphalaroides* Kunth; **Location:** country: Ecuador; stateProvince: Chimborazo; verbatimLocality: Camino a Yaruquies, aproximadamente 15 km a la izquierda, comunidad Cacha Chuyu; verbatimElevation: 3600 m; locationRemarks: Camino a Yaruquies, aproximadamente 15 km a la izquierda, comunidad Cacha Chuyu, 01°40’S 78° 45’W, 3600 m, bosque de *Pinusradiata* sobre páramo; georeferenceProtocol: label; **Event:** eventDate: 19-20 Jul 1993; **Record Level:** basisOfRecord: PreservedSpecimen
- **Type status:** Other material. **Occurrence:** catalogNumber: 22ECU-AMA22 (UPOS); recordNumber: 22; recordedBy: A. Morales Alonso, P. Jiménez Mejías, I. Masa Iranzo & E. Sánchez; occurrenceID: 22ECU-AMA22 (UPOS); **Taxon:** scientificName: *Carexphalaroides* Kunth; **Location:** country: Ecuador; stateProvince: Loja; verbatimLocality: Cerca de Ramos Urku, carretera Loja-Cuenca; verbatimElevation: 2900 m; locationRemarks: Cerca de Ramos Urku, carretera Loja-Cuenca, 03°40.5102'S 079°16.0522'W, 2900 m, pastizal; georeferenceProtocol: GPS; **Identification:** identifiedBy: P. Jiménez-Mejías; dateIdentified: 2022; **Event:** eventDate: Jul-28-2022; **Record Level:** basisOfRecord: PreservedSpecimen
- **Type status:** Other material. **Occurrence:** catalogNumber: 28ECU-AMA22 (HUTI, UPOS); recordNumber: 28; recordedBy: A. Morales Alonso, P. Jiménez Mejías, I. Masa Iranzo & E. Sánchez; occurrenceID: 28ECU-AMA22 (HUTI, UPOS); **Taxon:** scientificName: *Carexphalaroides* Kunth; **Location:** country: Ecuador; stateProvince: Loja; verbatimLocality: Cerca de Ońa; verbatimElevation: 2839 m; locationRemarks: Loja. Cerca de Oña, 03°30.5420'S 079°10.3658'W, 2839 m, matorral degradado sobre suelos arcillosos; georeferenceProtocol: GPS; **Identification:** identifiedBy: P. Jiménez-Mejías; dateIdentified: 2022; **Event:** eventDate: Jul-28-2022; **Record Level:** basisOfRecord: PreservedSpecimen
- **Type status:** Other material. **Occurrence:** catalogNumber: 92ECU-AMA22 (UPOS); recordNumber: 92; recordedBy: A. Morales Alonso, P. Jiménez Mejías & I. Masa Iranzo; occurrenceID: 92ECU-AMA22 (UPOS); **Taxon:** scientificName: *Carexphalaroides* Kunth; **Location:** country: Ecuador; stateProvince: Pichincha; verbatimLocality: Subida al cerro Corazón; verbatimElevation: 3837 m; locationRemarks: Pichincha. Subida al cerro Corazón, 00°34.0922'S 078°39.9008'W, 3837 m, borde camino en matorral; georeferenceProtocol: GPS; **Identification:** identifiedBy: P. Jiménez-Mejías; dateIdentified: 2022; **Event:** eventDate: 4 Aug 22; **Record Level:** basisOfRecord: PreservedSpecimen
- **Type status:** Other material. **Occurrence:** catalogNumber: 94ECU-AMA22 (HUTI, UPOS); recordNumber: 94; recordedBy: A. Morales Alonso, P. Jiménez Mejías & I. Masa Iranzo; occurrenceID: 94ECU-AMA22 (HUTI, UPOS); **Taxon:** scientificName: *Carexphalaroides* Kunth; **Location:** country: Ecuador; stateProvince: Pichincha; verbatimLocality: Subida al cerro Corazón; verbatimElevation: 3805 m; locationRemarks: Pichincha. Subida al cerro Corazón, 00°34.1401'S 078°39.8770'W, 3805 m, pajonal con matorral, en el camino y borde de camino; georeferenceProtocol: GPS; **Identification:** identifiedBy: P. Jiménez-Mejías; dateIdentified: 2022; **Event:** eventDate: 4 Aug 22; **Record Level:** basisOfRecord: PreservedSpecimen
- **Type status:** Other material. **Occurrence:** catalogNumber: 147ECU-AMA22 (UPOS); recordNumber: 147; recordedBy: A. Morales Alonso, P. Jiménez Mejías & I. Masa Iranzo; occurrenceID: 147ECU-AMA22 (UPOS); **Taxon:** scientificName: *Carexphalaroides* Kunth; **Location:** country: Ecuador; stateProvince: Pichincha; locality: Rucu-Pichincha; verbatimLocality: Camino desde estación final del teleférico (cerro Cruz Loma) al Rucu-Pichincha; verbatimElevation: 4078 m; locationRemarks: Pichincha. Camino desde estación final del teleférico (cerro Cruz Loma) al Rucu-Pichincha, 00°11.0232'S 078°32.3160'W, 4078 m, borde de camino en pajonal; georeferenceProtocol: GPS; **Identification:** identifiedBy: P. Jiménez-Mejías; dateIdentified: 2022; **Event:** eventDate: 13 Aug 22; **Record Level:** basisOfRecord: PreservedSpecimen
- **Type status:** Other material. **Occurrence:** catalogNumber: 79ECU-AMA22 (UPOS); recordNumber: 74; recordedBy: A. Morales Alonso, P. Jiménez Mejías & I. Masa Iranzo; occurrenceID: 79ECU-AMA22 (UPOS); **Taxon:** scientificName: *Carexphalaroides* Kunth; **Location:** country: Ecuador; stateProvince: Tungurahua; locality: National Park Llanganates; verbatimLocality: Carretera del Parque Nacional Llanganates; verbatimElevation: 3577 m; locationRemarks: Tungurahua. Carretera del Parque Nacional Llanganates, 01°04.8339'S 078°24.5341'W, 3577 m, pradera; georeferenceProtocol: GPS; **Identification:** identifiedBy: P. Jiménez-Mejías; dateIdentified: 2022; **Event:** eventDate: 2 Aug 22; **Record Level:** basisOfRecord: PreservedSpecimen
- **Type status:** Other material. **Occurrence:** catalogNumber: 74ECU-AMA22 (UPOS); recordNumber: 79; recordedBy: A. Morales Alonso, P. Jiménez Mejías & I. Masa Iranzo; occurrenceID: 74ECU-AMA22 (UPOS); **Taxon:** scientificName: *Carexphalaroides* Kunth; **Location:** country: Ecuador; stateProvince: Tungurahua; locality: National Park Llanganates; verbatimLocality: Carretera del Parque Nacional Llanganates; verbatimElevation: 3617 m; locationRemarks: Tungurahua. Carretera Parque Nacional Llanganates, 01°04.6880'S 078°25.6385'W, 3617 m, pastizal; georeferenceProtocol: GPS; **Identification:** identifiedBy: P. Jiménez-Mejías; dateIdentified: 2022; **Event:** eventDate: 2 Aug 22; **Record Level:** basisOfRecord: PreservedSpecimen

#### Taxon discussion

*Carexphalaroides* is a Neotropical endemic widely distributed from northern Patagonia to Colombia and Venezuela, with an isolated occurrence known in Guatemala (Jiménez-Mejías et al. 2018). It belongs to sect. Junciformes Kük. (subg. Psyllophorae (Degl.) Peterm.), one of the few *Carex* groups with a remarkably relatively high species diversity in South America (Benítez-Benítez et al. 2021a). *Carexphalaroides* is an anomalous member of sect. Junciformes from a morphological point of view as its inflorescence is constituted by several spikes (instead of a solitary one in the remainder of the section) (Benítez-Benítez et al. 2021a, Morales-Alonso et al., in prep.).

Previously known from just a handful locations in Ecuador, our field campaign has resulted in the collection of several additional populations; thus, *Carexphalaroides* seems to be more widely distributed along Ecuadorian high Andean grasslands. However, to date, it has been collected just a few times (see comments under General considerations at the end of the manuscript), as depicted by its scarce representation in herbaria (just one additional specimen from Ecuador: MO-5869195, Imbabura, Cotacachi-Cayapas, after revising the full collections of K, NY, MO and US).

### Carex punicola

D.B.Poind., Jim.Mejías & M.Escudero, Phytotaxa 291(4): 292 (2017)

A7FFE55C-5620-55DD-88A8-0542F9EDA1BE

#### Materials

- **Type status:** Other material. **Occurrence:** catalogNumber: QCA 36443, QCNE 140268; recordNumber: 55330; recordedBy: S. Lægaard; occurrenceID: QCA 36443, QCNE 140268; **Taxon:** scientificName: *Carexpunicola* D.B.Poind; **Location:** country: Ecuador; stateProvince: Bolívar; locality: Salinas-Los Arenales; verbatimLocality: Km 6 on road Salinas-Los Arenales; verbatimElevation: 4000 m; locationRemarks: Bolivar. Km 6 on road Salinas-Los Arenales, 01°22'S 79°00'W, 4000 m, grass-páramo and open rocks; georeferenceProtocol: label; **Event:** eventDate: Oct-02-1985; **Record Level:** basisOfRecord: PreservedSpecimen
- **Type status:** Other material. **Occurrence:** catalogNumber: QCA 36467; recordNumber: 71002; recordedBy: S. Lægaard; occurrenceID: QCA 36467; **Taxon:** scientificName: *Carexpunicola* D.B.Poind; **Location:** country: Ecuador; stateProvince: Chimborazo; verbatimLocality: Páramo above Azul along road to Osogochi; verbatimElevation: 4000-4200 m; locationRemarks: Chimborazo. Páramo above Azul along road to Osogochi, 02°18'S 78°42’W, 4000-4200 m, grass-páramo; georeferenceProtocol: label; **Event:** eventDate: 26 Apr 1988; **Record Level:** basisOfRecord: PreservedSpecimen
- **Type status:** Other material. **Occurrence:** catalogNumber: QCA 36404, QCNE 137371; recordNumber: 19590; recordedBy: S. Lægaard; occurrenceID: QCA 36404, QCNE 137371; **Taxon:** scientificName: *Carexpunicola* D.B.Poind; **Location:** country: Ecuador; stateProvince: Chimborazo; verbatimLocality: 7 km along páramo road from new road Ambato-Guaranda towards Carihuairazo; verbatimElevation: 4000-4200 m; locationRemarks: Chimborazo. 7 km along páramo road from new road Ambato-Guaranda towards Carihuairazo, 01°24’S 78°48’W, 4000-4200 m, dry gravelly soil; georeferenceProtocol: label; **Event:** eventDate: Feb-15-1999; **Record Level:** basisOfRecord: PreservedSpecimen
- **Type status:** Other material. **Occurrence:** catalogNumber: 82ECU-AMA22 (HUTI, UPOS); recordNumber: 82; recordedBy: A. Morales Alonso, P. Jiménez Mejías, & I. Masa Iranzo; occurrenceID: 82ECU-AMA22 (HUTI, UPOS); **Taxon:** scientificName: *Carexpunicola* D.B.Poind; **Location:** country: Ecuador; stateProvince: Pichincha; locality: National Park Cotopaxi; verbatimLocality: Carretera Parque Nacional Cotopaxi, cerca refugio Tombopaxi, entrada Norte; verbatimElevation: 3691 m; locationRemarks: Pichincha. Carretera Parque Nacional Cotopaxi, cerca refugio Tombopaxi, entrada Norte 00°34.0919'S 078°26.6518'W, 3691 m, páramo, pastizal seco; georeferenceProtocol: GPS; **Identification:** identifiedBy: P. Jiménez-Mejías; dateIdentified: 2022; **Event:** eventDate: 3 Aug 2022; **Record Level:** basisOfRecord: PreservedSpecimen

#### Taxon discussion

*Carexpunicola* was so far known as a Central Andean element, reported from Peru, Bolivia and Argentina (Wheeler 1988, Poindexter et al. 2017). This species is the only Southern Hemisphere taxon from section Acrocystis Dumort., a mostly Holarctic group greatly diversified in North America. *Carexpunicola* is a diminutive acaulescent sedge from dry soils (Fig. 1D,d). Interestingly, there is an unusually high number of acaulescent *Carex* species in South America, which suggests a possible evolutionary trend (Jiménez-Mejías et al. 2021a).

Here, we present the first records for Ecuador, which constitutes the new northernmost limit for the species.

### Carex roalsoniana

Jim.Mejías & M.Escudero, Phytotaxa 260: 186 (2016)

A0B58339-8AED-55F1-956A-8C1F974460B8

#### Materials

- **Type status:** Other material. **Occurrence:** catalogNumber: 55aECU-AMA22 (HUTI, UPOS); recordNumber: 55a; recordedBy: A. Morales Alonso, P. Jiménez Mejías, I. Masa Iranzo & E. Sánchez; occurrenceID: 55aECU-AMA22 (HUTI, UPOS); **Taxon:** scientificName: *Carexroalsoniana* Jim.Mejías & M.Escudero; **Location:** country: Ecuador; stateProvince: Azuay; locality: National Park Cajas; verbatimLocality: Laguna de Llaviucu; verbatimElevation: 3156 m; locationRemarks: Azuay. Laguna de Llaviucu, Parque Nacional Cajas, 02°05.6385'S 079°08.9762'W, 3156 m, borde de camino en bosque montano; georeferenceProtocol: GPS; **Identification:** identifiedBy: P. Jiménez-Mejías; dateIdentified: 2022; **Event:** eventDate: Jul-31-2022; **Record Level:** basisOfRecord: PreservedSpecimen

#### Taxon discussion

A recently-described species endemic from the northern Andes in Ecuador and Peru (Jiménez-Mejías and Escudero 2016, Jiménez-Mejías et al., under review). As *C.lepida* (see comments above), it belongs to sect. Wheelerianae.

In Ecuador, it was only known from the type locality also in Azuay Province, at Sevilla de Oro Municipality (Jiménez-Mejías and Escudero 2016), on the Eastern Cordillera. This constitutes the second record of the species in Ecuador and the first in the Western Cordillera. In the collected locality it grows together with the closely-related *C.lepida*, with which it is seemingly hybridising (see above).

### Carex sodiroi

Kük., Beibl. Bot. Jahrb. Syst. 78: 7 (1904)

A02E8832-2D10-51EA-A18B-8847AE092EDA

#### Materials

- **Type status:** Other material. **Occurrence:** catalogNumber: 121 ECU-AMA22 (HUTI, UPOS); recordNumber: 121; recordedBy: Morales Alonso, A., Jiménez Mejías, P., Masa Iranzo, I & Oña, P; occurrenceID: 121 ECU-AMA22 (HUTI, UPOS); **Taxon:** scientificName: *Carexsodiroi* Kük; **Location:** country: Ecuador; stateProvince: Pichincha; locality: Reserva Geobotánica Pululahua; verbatimLocality: carretera desde Moraspungo; locationRemarks: Pichincha. Reserva Geobotánica Pululahua, carretera desde Moraspungo, 00°02.1872'N 078°30.5291'W, 2749 m, bosque montano alterado en taludes al borde de la carretera; georeferenceProtocol: GPS; **Identification:** identifiedBy: P. Jiménez-Mejías; dateIdentified: 2022; **Event:** eventDate: 8 Aug 2022; **Record Level:** basisOfRecord: PreservedSpecimen
- **Type status:** Other material. **Occurrence:** catalogNumber: 122, 125, 126 ECU-AMA22 (HUTI, UPOS).; recordNumber: 122, 125, 126; recordedBy: Morales Alonso, A., Jiménez Mejías, P., Masa Iranzo, I & Oña, P.; occurrenceID: 122, 125, 126 ECU-AMA22 (HUTI, UPOS).; **Taxon:** scientificName: *Carexsodiroi* Kük; **Location:** country: Ecuador; stateProvince: Pichincha; locality: Reserva Geobotánica Pululahua; verbatimLocality: base Cerro Pan de Azúcar; locationRemarks: Pichincha. Reserva Geobotánica Pululahua, base Cerro Pan de Azúcar, 00°03.1237'N 078°29.2568'W - 00°03.5315'N 078°29.6173'W, 2662-2428 m, bosque montano bajo, camino en formación cerrada de "culunco"; georeferenceProtocol: GPS; **Identification:** identifiedBy: P. Jiménez-Mejías; dateIdentified: 2022; **Event:** eventDate: 8 Aug 2022; **Record Level:** basisOfRecord: PreservedSpecimen
- **Type status:** Other material. **Occurrence:** catalogNumber: 133 ECU-AMA22 (HUTI, UPOS).; recordNumber: 133; recordedBy: Morales Alonso, A., Jiménez Mejías, P., Masa Iranzo, I & Oña, P; occurrenceID: 133 ECU-AMA22 (HUTI, UPOS).; **Taxon:** scientificName: *Carexsodiroi* Kük; **Location:** country: Ecuador; stateProvince: Pichincha; locality: Reserva Geobotánica Pululahua; verbatimLocality: base Cerro Pondoña; locationRemarks: Pichincha. Reserva Geobotánica Pululahua, base Cerro Pondoña, 00°03.2663'N 078°30.1839'W, 2335 m, remanente de bosque en borde de camino abierto; georeferenceProtocol: GPS; **Identification:** identifiedBy: P. Jiménez-Mejías; dateIdentified: 2022; **Event:** eventDate: 8 Aug 2022; **Record Level:** basisOfRecord: PreservedSpecimen
- **Type status:** Other material. **Occurrence:** catalogNumber: 136 ECU-AMA22 (HUTI, UPOS).; recordNumber: 136; recordedBy: Morales Alonso, A., Jiménez Mejías, P., Masa Iranzo, I & Oña P.; occurrenceID: 136 ECU-AMA22 (HUTI, UPOS).; **Taxon:** scientificName: *Carexsodiroi* Kük; **Location:** country: Ecuador; stateProvince: Pichincha; locality: Reserva Geobotánica Pululahua. Quito-Gualea. El Pahuma; locationRemarks: Pichincha. Quito-Gualea. El Pahuma, 00°01.5696'N 078°37.9463'W, 1979 m, remanente de bosque montano, en pastizal al lado de la carretera; georeferenceProtocol: GPS; **Identification:** identifiedBy: P. Jiménez-Mejías; dateIdentified: 2022; **Event:** eventDate: 10 Aug 2022; **Record Level:** basisOfRecord: PreservedSpecimen
- **Type status:** Other material. **Occurrence:** catalogNumber: QCA 36711; recordNumber: 1847; recordedBy: C.E. Cerón, G. Benavídez & L. Velásquez; occurrenceID: QCA 36711; **Taxon:** scientificName: *Carexsodiro*i Kük; **Location:** country: Ecuador; stateProvince: Pichincha; locality: Reserva Geobotánica Pululahua; verbatimLocality: camino que bordea todo el Pondoña; locationRemarks: Pichincha. Parroqua Calacalí, Reserva Geobotánica Pululahua, camino que bordea todo el Pondoña; georeferenceProtocol: label; **Event:** eventDate: 8 Aug 1987; **Record Level:** basisOfRecord: PreservedSpecimen

#### Taxon discussion

*Carexsodiroi* is a poorly known Ecuadorian endemic species. It belongs to the problematic Decora Clade (subg. Carex) as part of sect. Indicae *sensu lato* (see comments under *C.aztecica*) (Fig. 1E,e) .

*Carexsodiroi* was reported in the Ecuadorian Red List as critically endangered (CR) because of the lack of recent collections. However, this is probably not due to a true rarity of the species, but to the poor taxonomic circumscription of *C.sodiroi*. Section Indicae in Tropical America has an obscure taxonomy. A number of taxa have been described, based on little material and within a local scale (e.g. *C.culmenicola* Steyerm. Steyermark (1951)), *C.regnelliana* Boeck. (Boeckeler (1888)). The whole variation of the species in the field remains understudied and, thus, the taxonomic limits are blurred by specimens that does not fit the characteristics and measurements that are presented in the available descriptions. *Carexsodiroi* is clearly affected by this lack of understanding. The only description associated with the name is Kükenthal (1904), which is in turn based just on the original collection. To date, no additional study has accounted for this taxon.

In our fieldwork campaign across the western slopes of the Western Cordillera in Pichincha, in forest remnants near to where the original collection of *C.sodiroi* was performed, we found plants that clearly match the diagnostic characteristics of *C.sodiroi*’s type, allowing its distinction from the syntopic and closely-related *C.polystachya*: terminal clusters of spikes densely packed with many flowers, concealing the branch axis vs. with less flowers and lax, with the branch axis exposed; and glumes dark orange-brown vs. glumes lighter, pale reddish-brown, orange, stramineous or hyaline. Moreover, in Pululahua Geobotanic Reserve, we also identified individuals morphologically intermediate (124ECUAMA22 and 130ECUAMA22; HUTI, UPOS) between the co-occurring *C.polystachya* and *C.porrecta* Reznicek and Camelb., another species of sect. Indicae, whose morphology also approached the type of *C.sodiroi*. This makes us think that, perhaps, *C.sodiroi* is a local established hybrid between these two species. Further biosystematics studies are needed to figure out the taxonomic status of *C.sodiroi* and its systematic relationships with the other South American sect. Indicae species.

### Carex subsacculata

(G.A.Wheeler & Goetgh.) J.R.Starr, Bot. J. Linn. Soc. 179: 36 (2015)

7825814C-112A-5FE0-93A6-89FE1ABA30C9

=*Unciniasubsacculata* G.A.Wheeler & Goetgh., Aliso 14: 145 (1995).

#### Materials

- **Type status:** Other material. **Occurrence:** catalogNumber: 146 ECU-AMA22 (HUTI, UPOS); recordNumber: 146; recordedBy: Morales Alonso, A., Jiménez Mejías, P., Masa Iranzo, I; occurrenceID: 146 ECU-AMA22 (HUTI, UPOS); **Taxon:** scientificName: Carexsubsacculata (G.A.Wheeler & Goetgh.) J.R.Starr; **Location:** country: Ecuador; stateProvince: Pichincha; locality: Reserva Biológica Yanacocha; verbatimLocality: sendero Andean Snipe; verbatimElevation: 3620-3796 m; locationRemarks: Pichincha. Reserva Biológica Yanacocha, sendero Andean Snipe, 00°07.3179'S 078°35.1625'W, bosque alto *Polylepis* spp. Sobre abundante suelo mesófilo de musgos; georeferenceProtocol: GPS; **Identification:** identifiedBy: P. Jiménez-Mejías; dateIdentified: 2022; **Event:** eventDate: 10 Aug 2022; **Record Level:** basisOfRecord: PreservedSpecimen

#### Taxon discussion

The last of the sect. Uncinia species in our account (see *C.ecuadorensis*).

*Carexsubsacculata* was reported in the Ecuadorian Red List (as *Unciniasubsacculata*) as vulnerable (VU), despite being known from a single locality, it was located in a protected area (Yanacocha Biological Reserve) in the Province of Pichincha. We confirm the persistence and healthy conservation status of this population despite the recent eruptions of the nearby Pichincha Volcano (last eruption in 2002). *Carexsubsacculata* forms an abundant population in *Polylepis* forest understorey, at the very top of the Andean Snipe track in the Reserve (Fig. 1 F1,F2,f) .

### Carex via-incaica

Jim.Mejías & Roalson, Phytotaxa 266: 21 (2016)

FE23F6FE-0731-5FCD-BF24-BC37B8BDF052

#### Materials

- **Type status:** Other material. **Occurrence:** catalogNumber: P.M.Ramsay & P.J.Marrow-Smith 358 (K); recordNumber: 358; recordedBy: P.M.Ramsay & P.J.Marrow-Smith; occurrenceID: P.M.Ramsay & P.J.Marrow-Smith 358 (K); **Taxon:** scientificName: *Carexvia-incaica* Jim.Mejías & Roalson; **Location:** country: Ecuador; stateProvince: Chimborazo; locality: Páramo de los Altares; locationRemarks: Chimborazo. Páramo de los Altares, 01°40'S 78°24'W, 4000 m; georeferenceProtocol: label; **Event:** eventDate: Sep-03-1987; **Record Level:** basisOfRecord: PreservedSpecimen
- **Type status:** Other material. **Occurrence:** catalogNumber: QCA 36470; recordNumber: 71693; recordedBy: S. Lægaard; occurrenceID: QCA 36470; **Taxon:** scientificName: *Carexvia-incaica* Jim.Mejías & Roalson; **Location:** country: Ecuador; stateProvince: Chimborazo; locality: Arenales de Palmiras; locationRemarks: Chimborazo. Arenales de Palmiras, 02°02'S 78°45'W, 3200-3300 m; georeferenceProtocol: label; **Event:** eventDate: Jun-26-1988; **Record Level:** basisOfRecord: PreservedSpecimen
- **Type status:** Other material. **Occurrence:** catalogNumber: QCA 36566, QCA 36569; recordNumber: 95-9; 98-14; recordedBy: P. Sklenář & V. Kostečková; occurrenceID: QCA 36566, QCA 36569; **Taxon:** scientificName: *Carexvia-incaica* Jim.Mejías & Roalson; **Location:** country: Ecuador; stateProvince: Chimborazo; locality: El Altar; verbatimLocality: N side; locationRemarks: Chimborazo. El Altar, N side, 1°41'S 78°24'W, 4200 m, humid cushion (super)páramo with scattered bunchgrasses on the ridge below the Canoningo peak; georeferenceProtocol: label; **Event:** eventDate: 20 Aug 1995; **Record Level:** basisOfRecord: PreservedSpecimen
- **Type status:** Other material. **Occurrence:** catalogNumber: QCA 36517; recordNumber: 125-3; recordedBy: P. Sklenář & V. Kostečková; occurrenceID: QCA 36517; **Taxon:** scientificName: *Carexvia-incaica* Jim.Mejías & Roalson; **Location:** country: Ecuador; stateProvince: Imbabaura; locality: Cotacachi volcano; verbatimLocality: south-western side of the SE ridge; locationRemarks: Imbabura. Cotacachi volcano, south-western side of the SE ridge, 00°21'N 78°21'W, 4300 m, shrub and cushion páramo; georeferenceProtocol: label; **Event:** eventDate: Sep-09-1995; **Record Level:** basisOfRecord: PreservedSpecimen
- **Type status:** Other material. **Occurrence:** catalogNumber: QCA 36434; recordNumber: 53863; recordedBy: S. Laegaard; occurrenceID: QCA 36434; **Taxon:** scientificName: *Carexvia-incaica* Jim.Mejías & Roalson; **Location:** country: Ecuador; stateProvince: Napo; verbatimLocality: Road Pifo-Papallacta, N of the antennas at Paso La Virgen; locationRemarks: Napo. Road Pifo-Papallacta, N of the antennas at Paso La Virgen, 00°17'S 78°11'W, 4250-4400 m; georeferenceProtocol: label; **Event:** eventDate: 13 March 1985; **Record Level:** basisOfRecord: PreservedSpecimen
- **Type status:** Other material. **Occurrence:** catalogNumber: QCA 36550; recordNumber: 3550; recordedBy: P. Sklenář & V. Kostečková; occurrenceID: QCA 36550; **Taxon:** scientificName: *Carexvia-incaica* Jim.Mejías & Roalson; **Location:** country: Ecuador; stateProvince: Napo; locality: Antisana volcano; verbatimLocality: NE side; locationRemarks: Napo. Antisana volcano, NE side, 00°27'S 78°08'W, 4200-4300 m, superpáramo vegetation on the wet soil below rocky escarpments; georeferenceProtocol: label; **Event:** eventDate: 19 Aug 1997; **Record Level:** basisOfRecord: PreservedSpecimen
- **Type status:** Other material. **Occurrence:** catalogNumber: QCA 36322; recordNumber: 001; recordedBy: Freddy Bravo; occurrenceID: QCA 36322; **Taxon:** scientificName: *Carexvia-incaica* Jim.Mejías & Roalson; **Location:** country: Ecuador; stateProvince: Napo-Pichincha; locality: Páramo de Guamaní, Paso de La Virgen; locationRemarks: Napo-Pichincha. Páramo de Guamaní, Paso de La Virgen, 0°22'S 78°12'W, 4000 m; georeferenceProtocol: label; **Event:** eventDate: 17 Dec 1983; **Record Level:** basisOfRecord: PreservedSpecimen
- **Type status:** Other material. **Occurrence:** catalogNumber: QCA 36433; recordNumber: 53876; recordedBy: S. Laegaard; occurrenceID: QCA 36433; **Taxon:** scientificName: *Carexvia-incaica* Jim.Mejías & Roalson; **Location:** country: Ecuador; stateProvince: Pichincha; locality: Atacazo volcano; locationRemarks: Pichincha. Atacazo volcano, 00°2'S 78°37'W, 4250-4450 m, páramo; georeferenceProtocol: label; **Event:** eventDate: 15 March 1985; **Record Level:** basisOfRecord: PreservedSpecimen
- **Type status:** Other material. **Occurrence:** catalogNumber: QCA 36462; recordNumber: 70522; recordedBy: S. Lægaard; occurrenceID: QCA 36462; **Taxon:** scientificName: *Carexvia-incaica* Jim.Mejías & Roalson; **Location:** country: Ecuador; stateProvince: Pichincha; locality: Cayambe volcano; verbatimLocality: along road to Refugio; locationRemarks: Pichincha. Cayambe volcano, along road to Refugio, 00°04'S 77°57'W, 4300 m, muddy flats in swamp; georeferenceProtocol: label; **Event:** eventDate: Mar-02-1988; **Record Level:** basisOfRecord: PreservedSpecimen
- **Type status:** Other material. **Occurrence:** catalogNumber: QCA 36317; recordNumber: 3360; recordedBy: H. Balslev, J. Brandbyge and L. Coloma; occurrenceID: QCA 36317; **Taxon:** scientificName: *Carexvia-incaica* Jim.Mejías & Roalson; **Location:** country: Ecuador; stateProvince: Pichincha; locality: National Park Cotopaxi, Cotopaxi volcano; verbatimLocality: N slope; locationRemarks: Pichincha. Parque Nacional Cotopaxi, Cotopaxi volcano, N slope, 00°40'S 78°30'W, 4100-4300 m, páramo and rocky slopes; georeferenceProtocol: label; **Event:** eventDate: Oct-28-1982; **Record Level:** basisOfRecord: PreservedSpecimen
- **Type status:** Other material. **Occurrence:** catalogNumber: QCA 116027; recordNumber: 51316; recordedBy: S. Lægaard; occurrenceID: QCA 116027; **Taxon:** scientificName: *Carexvia-incaica* Jim.Mejías & Roalson; **Location:** country: Ecuador; stateProvince: Pichincha; locality: Páramo Guamaní; verbatimLocality: close to Paso La Virgen; locationRemarks: Pichincha. Páramo Guamaní, close to Paso La Virgen, 00°20'S 78°13'W, 4050 m, moist páramo and springbogs; georeferenceProtocol: label; **Event:** eventDate: Feb-08-1984; **Record Level:** basisOfRecord: PreservedSpecimen
- **Type status:** Other material. **Occurrence:** catalogNumber: QCA 36421; recordNumber: 52147; recordedBy: S. Lægaard; occurrenceID: QCA 36421; **Taxon:** scientificName: *Carexvia-incaica* Jim.Mejías & Roalson; **Location:** country: Ecuador; stateProvince: Pichincha; locality: Paso de la Virgen; verbatimLocality: 2 km south, road Quito-Baeza; locationRemarks: Pichincha. Paso de la Virgen, 2 km south, road Quito-Baeza, 00°20'S 78°13'W, 4000-4200 m, on rock with seeping water; georeferenceProtocol: label; **Event:** eventDate: 19-20 May 1984; **Record Level:** basisOfRecord: PreservedSpecimen
- **Type status:** Other material. **Occurrence:** catalogNumber: QCA 36551; recordNumber: 3610; recordedBy: P. Sklenář & V. Kostečková; occurrenceID: QCA 36551; **Taxon:** scientificName: *Carexvia-incaica* Jim.Mejías & Roalson; **Location:** country: Ecuador; stateProvince: Tungurahua; locality: Cerro Hermoso; verbatimLocality: SW ridge; locationRemarks: Tungurahua. Cerro Hermoso, SW ridge, 1°14'S 78°18'W, 4100 m, transition from the grass/bamboo páramo to lower superpáramo with shrubs of *Lorica* spp; georeferenceProtocol: label; **Event:** eventDate: Sep-06-1997; **Record Level:** basisOfRecord: PreservedSpecimen
- **Type status:** Other material. **Occurrence:** catalogNumber: P.M.Ramsay & P.J.Marrow-Smith 298 (K); recordNumber: 298; recordedBy: P.M.Ramsay & P.J.Marrow-Smith; occurrenceID: P.M.Ramsay & P.J.Marrow-Smith 298 (K); **Taxon:** scientificName: *Carexvia-incaica* Jim.Mejías & Roalson; **Location:** country: Ecuador; stateProvince: Tungurahua; locality: Tungurahua volcano; verbatimLocality: N slopes; locationRemarks: Tungurahua. N slopes of Volcan Tungurahua, 01°29'S 78°23'W, páramo; georeferenceProtocol: label; **Event:** eventDate: 30 Aug 1987; **Record Level:** basisOfRecord: PreservedSpecimen
- **Type status:** Other material. **Occurrence:** catalogNumber: QCA 234420; recordNumber: 298; recordedBy: P.M.Ramsay & P.J.Marrow-Smith; occurrenceID: QCA 234420; **Taxon:** scientificName: *Carexvia-incaica* Jim.Mejías & Roalson; **Location:** country: Ecuador; stateProvince: Tungurahua; locality: Tungurahua volcano; verbatimLocality: N slopes; locationRemarks: Tungurahua. Tungurahua volcano, N slopes, 01°29'S 78°23'W, 3900 m, páramo; georeferenceProtocol: label; **Event:** eventDate: 30 Aug 1987; **Record Level:** basisOfRecord: PreservedSpecimen

#### Taxon discussion

*Carexvia-incaica* was recently described from a single collection at the slopes of Rumiñahui Volcano in Cotopaxi Province (Jiménez-Mejías and Roalson 2016). It is one of the few northern South American species of the sect. Junciformes (subg. Psyllophorae), a group with its centres of origin and diversity in Patagonia (Benítez-Benítez et al. 2021a).

We present several records that greatly expand the range of this Ecuadorian endemic species. The high number of herbarium specimens at QCA and QCNE (stored as undetermined sedges) depicts the fact that this species has not remained overlooked nor undercollected, but just not understood.

### Hypericum acostanum

Steyerm. ex N. Robson, Bull. Brit. Mus. (Nat. Hist.), Botany Series 16: 27 (1987)

FC5BF111-D2D1-5597-9456-AD277810DC56

#### Materials

- **Type status:** Other material. **Occurrence:** catalogNumber: 1ECU-IMI22 (HUTI, MA); recordNumber: 1; recordedBy: I. Masa Iranzo, P. Jiménez Mejías, A. Morales Alonso & E. Sánchez; occurrenceID: 1ECU-IMI22 (HUTI, MA); **Taxon:** scientificName: *Hypericumacostanum* Steyerm. ex N. Robson; **Location:** country: Ecuador; stateProvince: Loja; verbatimLocality: Carretera entre Celica-Guachanamá; verbatimElevation: 2456 m; locationRemarks: Loja. Carretera entre Celica-Guachanamá, 04°04.1611'S 079°55.4277'W, -4,06935166666667 -79,923795, 2456 m, claro matorral; georeferenceProtocol: GPS; **Identification:** identifiedBy: I. Masa-Iranzo; dateIdentified: 2022; **Event:** eventDate: Jul-27-2022; **Record Level:** basisOfRecord: PreservedSpecimen
- **Type status:** Other material. **Occurrence:** catalogNumber: 6ECU-IMI22 (HUTI, MA); recordNumber: 6; recordedBy: I. Masa Iranzo, P. Jiménez Mejías, A. Morales Alonso & E. Sánchez; occurrenceID: 6ECU-IMI22 (HUTI, MA); **Taxon:** scientificName: *Hypericumacostanum* Steyerm. ex N. Robson; **Location:** country: Ecuador; stateProvince: Loja; verbatimLocality: Carretera entre Celica-Guachanamá; verbatimElevation: 2631 m; locationRemarks: Loja. Carretera entre Celica-Guachanamá, 04°03.7098'S 079°54.2242'W, -4,06183 -79,9037366666667, 2631 m, matorral-pastizal degradado; georeferenceProtocol: GPS; **Identification:** identifiedBy: I. Masa-Iranzo; dateIdentified: 2022; **Event:** eventDate: Jul-27-2022; **Record Level:** basisOfRecord: PreservedSpecimen
- **Type status:** Other material. **Occurrence:** catalogNumber: QCNE 5612; recordNumber: 50236; recordedBy: J. Madsen; occurrenceID: QCNE 5612; **Taxon:** scientificName: *Hypericumacostanum* Steyerm. ex N. Robson; **Location:** country: Ecuador; stateProvince: Loja; locality: Vicinity of Loja; verbatimLocality: at road towards La Toma (Catamayo); verbatimElevation: 2200m; locationRemarks: Loja. Vicinity of Loja, at road towards La Toma (Catamayo). 04°00'S 79°13'W, 2200m, 19 Feb 1984, Disturbed xerophytic vegetation, locally more humid. Bush 20 cm high; georeferenceProtocol: label; **Event:** eventDate: Feb-19-1984; **Record Level:** basisOfRecord: PreservedSpecimen

#### Taxon discussion

**Figure 2.**
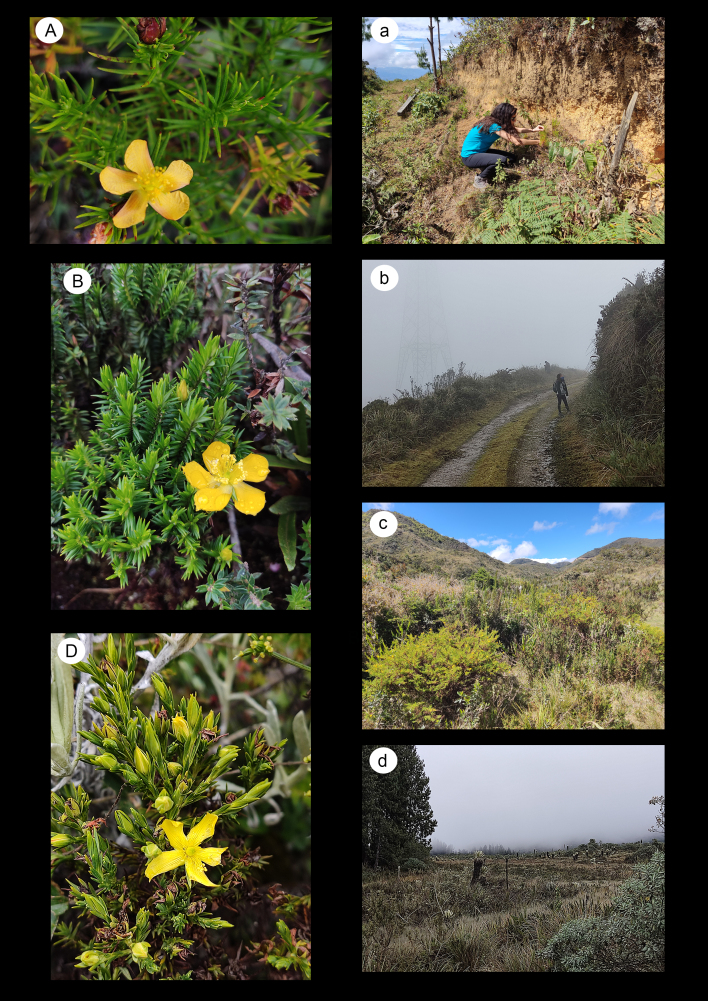
Representative Ecuadorian *Hypericum* species (capital letters) mentioned in this work and their habitats (small letters). *Hypericumacostanum*: **A & a**, Loja, Celica-Guachanamá road. *Hypericummatangense*: **B & b** Morona-Santiago, Páramo de Matanga. *Hypericumprietoi*: **c** Azuay, Oña-Morasloma road. *Hypericumsprucei*: **D & d** Carchi, Reserva Ecológica El Ángel.

*Hypericumacostanum* (Fig. 2A,a) is an endemic species of southern Ecuador. It belongs to the New World section Brathys (Mutis ex L.f.) Choisy, which has radiated in the Páramos (Nürk et al. 2013).

This species is listed as vulnerable (VU) in the Ecuadorian Red List. It is known from only two localities, collected in the 50s and 80s in the province of Loja, on the road between San Pedro and Portovelo and on the road between Celica-Guachanamá, respectively. We confirm its persistence in the second locality and provide an exact location with coordinates. In addition, after studying the QCNE herbarium material, we have re-assigned a specimen (voucher nº 5612) from the rovince of Loja to this species. Therefore, we have added a new locality to the distribution of *H.acostanum* in this rovince.

### Hypericum matangense

N. Robson, Bull. Brit. Mus. (Nat. Hist.), Botany Series 20: 17 (1990)

11B0D631-F1A8-503A-987A-CEF722654F93

#### Materials

- **Type status:** Other material. **Occurrence:** catalogNumber: 29ECU-IMI22 (HUTI, MA); recordNumber: 29; recordedBy: I. Masa Iranzo, P. Jiménez Mejías, A. Morales Alonso & E. Sánchez; occurrenceID: 29ECU-IMI22 (HUTI, MA); **Taxon:** scientificName: *Hypericummatangense* N. Robson; **Location:** country: Ecuador; stateProvince: Morona-Santiago; locality: Páramo de Matanga; verbatimLocality: carretera Sígsig-Gualaquiza; verbatimElevation: 3366 m; locationRemarks: Morona-Santiago. Páramo de Matanga, carretera Sígsig-Gualaquiza, 03°11.5409'S 078°47.3068'W -3,1924 -78,7885, 3366 m, Páramo con matorral; georeferenceProtocol: GPS; **Identification:** identifiedBy: I. Masa-Iranzo; dateIdentified: 2022; **Event:** eventDate: Jul-31-2022; **Record Level:** basisOfRecord: PreservedSpecimen

#### Taxon discussion

*Hypericummatangense* (Fig. 2B,b.) is an endemic plant from southern Ecuador. It belongs to section Brathys (Mutis ex L.f.) Choisy.

This species is listed as vulnerable (VU) in the Ecuadorian Red List. It is known onlyfrom the type specimen collected in 1980 in the province of Morona-Santiago. The size and status of this population, and its specific threats, are unknown. Our observation is the second known collection of this species, with coordinates from the only specimen found.

### Hypericum prietoi

N. Robson, Bull. Brit. Mus. (Nat. Hist.), Botany Series 16: 58 (1987)

604D1F14-54BB-54A4-B184-204772D1D8C6

#### Materials

- **Type status:** Other material. **Occurrence:** catalogNumber: 17ECU-IMI22 (HUTI, MA); recordNumber: 17; recordedBy: I. Masa Iranzo, P. Jiménez Mejías, A. Morales Alonso & E. Sánchez; occurrenceID: 17ECU-IMI22 (HUTI, MA); **Taxon:** scientificName: *Hypericumprietoi* N. Robson; **Location:** country: Ecuador; stateProvince: Azuay; verbatimLocality: Carretera Oña-Morasloma; verbatimElevation: 2822 m; locationRemarks: Azuay. Carretera Oña-Morasloma, 03 26.6385´S 079° 04.5871´W, 2822 m, matorral-pastizal; georeferenceProtocol: GPS; **Identification:** identifiedBy: I. Masa-Iranzo; dateIdentified: 2022; **Event:** eventDate: Jul-29-2022; **Record Level:** basisOfRecord: PreservedSpecimen

#### Taxon discussion

*Hypericumprietoi* (Fig. 2c) is an endemic species of southern Ecuador. It belongs to section Brathys (Mutis ex L.f.) Choisy.

This species is listed as endangered (EN) in the Ecuadorian Red List. Threats associated with its conservation status include overgrazing and burning. *Hypericumprietoi* has only been collected once at the type locality, in 1945. Our observation is the second known collection of the species. We provide the coordinates of the only specimen found.

### Hypericum sprucei

N. Robson, Bull. Brit. Mus. (Nat. Hist.), Botany Series 16: 65 (1987)

DAF03156-0F06-52FA-A3C5-3430FE368B48

#### Materials

- **Type status:** Other material. **Occurrence:** catalogNumber: QCA 50283; recordNumber: 212; recordedBy: X. Buitrón; occurrenceID: QCA 50283; **Taxon:** scientificName: *Hypericumsprucei* N. Robson; **Location:** country: Ecuador; stateProvince: Bolívar; verbatimLocality: Carretera Chimbo a Totoras; verbatimElevation: 3250 m; locationRemarks: Bolívar. Carretera Chimbo a Totoras, 3250 m; georeferenceProtocol: label; **Event:** eventDate: Feb-21-1987; **Record Level:** basisOfRecord: PreservedSpecimen

#### Taxon discussion

*Hypericumsprucei* (Fig. 2D,d) has been reported from a handful of provinces in Ecuador. It belongs to section Brathys (Mutis ex L.f.) Choisy.

Here, we present an additional record for a new province (Bolívar). This species is not included in the Ecuadorian Red List.

## Discussion

**General considerations about Ecuadorian *Carex***

Our work reveals a number of common problems in the knowledge of *Carex* in South America in general and in Ecuador in particular. Although Ecuador is one of the countries in tropical America with the most developed description of its flora, it is not free from the lack of taxonomic understanding of such a difficult group of plants as sedges.

On the one hand, despite the abundant collections in the Páramos promoted by the Ecuadorian government through research projects and vegetation inventories, the relative amount of sedges in the two main national herbaria (QCA, QCNE) is still low. Our field studies have shown that *Carex* can be among the most abundant plants in páramos, which highlights their ecological importance. However, only a few of the largest species from sect. Fecundae (i.e. *C.jamesonii* Boott, *C.lemanniana* Boott and *C.pichinchensis* Kunth) and *Uncinia* (*C.phleoides* Cav.) have been abundantly collected and are available in the QCA and QCNE collections. In particular, we found a large number of populations of *C.phalaroides* scattered throughout the cordillera, but only a handful of specimens in the herbaria we visited. Another example, and perhaps the most surprising for us, is the discovery of *C.punicola* for the first time in Ecuador. This species has been completely overlooked to date despite the fact that it can be a dominant part of the vegetation. In fact, in the population collected in the province of Pichincha, the plant was an abundant element of the sparse vegetation, extending over the lower part of the Ovejería puna and even occurring on such accessible places as the road shoulders of one of the main roads in Cotopaxi National Park. This neglect of *Carex* diversity is probably due to the combined effect of the small size of some of the species involved and the low interest of most naturalists in graminoids.

On the other hand, some species, such as *C.collumanthus* or *C.via-incaica*, were relatively well-represented in the herbaria studied despite their small size. This suggests that such species were probably abundant enough in a number of locations to have been overlooked during field collections. However, most of these specimens were missidentified or simply stored as indeterminate. This illustrates the problem of the lack of a comprehensive treatment for *Carex* in South America in general, and in Ecuador in particular. The lack of an accurate taxonomic treatment is probably what has prevented the formal description of *C.via-incaica* by other botanists before 2016, as there were already a number of vouchers in QCA or QCNE.

In conclussion, a greater effort in terms of taxonomic and systematic resources is needed before an accurate knowledge of the genus *Carex* can be achieved. As a result from this work, future changes in the number of native and endemic *Carex* species reported for Ecuador can be expected as progress is made towards a complete national checklist. This is of paramount importance since species of this genus, as already mentioned, can be abundant, or even dominant, elements of the páramo flora, and thus play a critical role in the ecological functioning of the habitats in which they thrive.

**General considerations about Ecuadorian *Hypericum***

Two species of *Hypericum* in Ecuador, *H.matangense* and *H.prietoi*, were described in 1990 and 1987, respectively and are only known from their type locality. No other records or additional collections are known to us since then. Our expedition seems to be the first discoverty of these species again. During our field campaign and despite our efforts to find other populations, we only found these two species again in their type localities. Apparently, both species have extremely small populations: we found only one specimen of each species. The distribution of these species should be carefully assessed. Similarly, *H.acostanum* has only been found in three localities since 1987 and we found it in low abundance in one of them.

*Hypericummatangense* and *H.acostanum* were listed as VU in the Ecuadorian Red List, while *H.prietoi* was listed as EN. Our observations suggest that these species may be critically endangered. If this is confirmed, the Red List status of *H.matangense* and *H.prietoi* will need to be updated and urgent conservation plans including *in situ* and *ex situ* measurements, will need to be implemented.

## Supplementary Material

XML Treatment for Carex acutata

XML Treatment for Carex aztecica

XML Treatment for Carex brehmeri

XML Treatment for Carex collumanthus

XML Treatment for Carex ecuadorensis

XML Treatment for Carex enneastachya

XML Treatment for Carex fecunda

XML Treatment for Carex goetghebeurii

XML Treatment for Carex lepida

XML Treatment for Carex lepida × roalsoniana

XML Treatment for Carex madida

XML Treatment for Carex melanocystis

XML Treatment for Carex phalaroides

XML Treatment for Carex punicola

XML Treatment for Carex roalsoniana

XML Treatment for Carex sodiroi

XML Treatment for Carex subsacculata

XML Treatment for Carex via-incaica

XML Treatment for Hypericum acostanum

XML Treatment for Hypericum matangense

XML Treatment for Hypericum prietoi

XML Treatment for Hypericum sprucei
